# PR/SET Domain Family and Cancer: Novel Insights from The Cancer Genome Atlas

**DOI:** 10.3390/ijms19103250

**Published:** 2018-10-19

**Authors:** Anna Sorrentino, Antonio Federico, Monica Rienzo, Patrizia Gazzerro, Maurizio Bifulco, Alfredo Ciccodicola, Amelia Casamassimi, Ciro Abbondanza

**Affiliations:** 1 Department of Precision Medicine, University of Campania “Luigi Vanvitelli”, Via L. De Crecchio, 80138 Naples, Italy; anna.sorrentino@unicampania.it; 2 Department of Science and Technology, University of Naples “Parthenope”, 80143 Naples, Italy; antonio.federico@igb.cnr.it (A.F.); alfredo.ciccodicola@igb.cnr.it (A.C.); 3 Institute of Genetics and Biophysics “Adriano Buzzati Traverso”, CNR, 80131 Naples, Italy; 4 Department of Environmental, Biological, and Pharmaceutical Sciences and Technologies, University of Campania “Luigi Vanvitelli”, 81100 Caserta, Italy; monica.rienzo@unicampania.it; 5 Department of Pharmacy, University of Salerno, 84084 Salerno, Italy; pgazzerro@unisa.it; 6 Department of Molecular Medicine and Medical Biotechnologies, University of Naples “Federico II”, 80131 Naples, Italy; maubiful@unina.it

**Keywords:** PRDM gene family, TCGA data analysis, somatic mutations, transcriptome profiling, human malignancies

## Abstract

The PR/SET domain gene family (PRDM) encodes 19 different transcription factors that share a subtype of the SET domain [Su(var)3-9, enhancer-of-zeste and trithorax] known as the PRDF1-RIZ (PR) homology domain. This domain, with its potential methyltransferase activity, is followed by a variable number of zinc-finger motifs, which likely mediate protein–protein, protein–RNA, or protein–DNA interactions. Intriguingly, almost all PRDM family members express different isoforms, which likely play opposite roles in oncogenesis. Remarkably, several studies have described alterations in most of the family members in malignancies. Here, to obtain a pan-cancer overview of the genomic and transcriptomic alterations of *PRDM* genes, we reanalyzed the Exome- and RNA-Seq public datasets available at The Cancer Genome Atlas portal. Overall, *PRDM2*, *PRDM3/MECOM*, *PRDM9*, *PRDM16* and *ZFPM2/FOG2* were the most mutated genes with pan-cancer frequencies of protein-affecting mutations higher than 1%. Moreover, we observed heterogeneity in the mutation frequencies of these genes across tumors, with cancer types also reaching a value of about 20% of mutated samples for a specific *PRDM* gene. Of note, *ZFPM1/FOG1* mutations occurred in 50% of adrenocortical carcinoma patients and were localized in a hotspot region. These findings, together with OncodriveCLUST results, suggest it could be putatively considered a cancer driver gene in this malignancy. Finally, transcriptome analysis from RNA-Seq data of paired samples revealed that transcription of *PRDMs* was significantly altered in several tumors. Specifically, *PRDM12* and *PRDM13* were largely overexpressed in many cancers whereas *PRDM16* and *ZFPM2/FOG2* were often downregulated. Some of these findings were also confirmed by real-time-PCR on primary tumors.

## 1. Introduction

The positive regulatory domain (PRDM) gene family, a subfamily of Kruppel-like zinc finger gene products, currently includes 19 members in humans [1,2,3,4]. The protein products of this family share a conserved N-terminal PR (PRDI-BF1-RIZ1 homologous) domain, which is structurally and functionally similar to the catalytic SET domain that defines a large group of histone methyltransferases (HMTs) [5,6,7,8]. So far, enzymatic activity has been experimentally demonstrated for only a few family members; otherwise, PRDM proteins (PRDMs) lacking intrinsic enzymatic activity are able to recruit histone-modifying enzymes to mediate their function. 

The PR domain is generally positioned at the protein N-terminal region and, with the exception of PRDM11, it is followed by repeated zinc fingers toward the C-terminus, potentially mediating sequence-specific DNA or RNA binding and protein–protein interactions [5,6,7,8,9] (Figure S1). Importantly, PRDMs have been established to tether transcription factors to target gene promoters by recognition of a specific DNA consensus sequence [10] or acting as non-DNA binding cofactors [11,12]. These features give PRDMs the ability to drive cell differentiation and to specify cell fate choice and, thus, contribute to many developmental processes [5,7,12,13,14].

A common characteristic of most *PRDM* genes is to express two main molecular variants, one lacking the PR domain (PR-minus isoform) but otherwise identical to the other PR-containing product (PR-plus isoform). These two isoforms, generated either by alternative splicing or alternative use of different promoters [7,8,15], play opposite roles, particularly in cancer. The full-length product PR-plus usually acts as a tumor suppressor, whereas the short isoform functions as an oncogene. This bivalent behavior has been tagged as ‘yin-yang’. The imbalance in favor of the PR-minus is found in many human malignancies and it can be due to inactivating mutations or silencing of the complete form and/or to increased expression of the PR-minus form [8].

*PRDM1* and *PRDM2* use alternative promoters to generate short isoforms lacking the PR domain, which show oncogenic properties. Increased levels of the short isoforms were reported in various cancer cell lines. A similar PR-less product was also described for *PRDM3/MECOM* (MDS1 and EVI1 complex locus), *PRDM16*, and *PRDM6*, thus suggesting that this ‘yin-yang’ expression pattern and its functional implications could be a hallmark of most, if not all, PRDMs [8].

Furthermore, several studies have described alterations (both mutations and/or gene expression changes) of most PRDMs in solid tumors and/or hematological malignancies [8]. For instance, frameshift mutations of microsatellite repeats within the *PRDM2* coding region are frequent events in various cancers. A recent study has described a frameshift mutation in the C-terminal region of PRDM2, affecting the (A)9 repeat within exon 8, as a microsatellite indel driver hotspot and as a driver mutation in microsatellite instability (MSI) colorectal cancer [15,16]. Notably, a similar frameshift mutation was found to occur in a mononucleotide repeat (A7) of *PRDM3/MECOM* gene in this cancer type [17]. Intriguingly, recent findings also indicate that PRDM2 methyltransferase is required for BRCA1-mediated genome maintenance [15,18]. Moreover, a significant reduction of *PRDM2* expression was observed in high-grade gliomas [19], and forced expression of *PRDM2* in glioma cell lines inhibits cell proliferation and increases apoptosis. This evidence strongly suggests a possible tumor suppressive role for PRDM2 [19]. Interestingly, PRDM9 HMTase activity is essential for meiotic DNA double-strand break formation at its binding sites [20,21]. Moreover, both PRDM1 and PRDM5 negatively modulate WNT/β-catenin signaling, a pathway involved in the occurrence of several cancers, including glioma and colorectal cancer [22,23].

This evidence suggests that PRDMs are involved in human cancer through modulation of several processes, such as epigenetic modifications, genetic reprogramming, inflammation, and metabolic homeostasis. 

To date, both mutations and altered expression have been reported for some *PRDMs* in specific cancer entities. However, our understanding of the role played by different PRDM family members in cancer is still limited by the lack of a systematic and comprehensive approach in deciphering the mutational status and the complete transcriptional profile of all the *PRDMs* across a large number of different cancer types.

Here, The Cancer Genome Atlas (TCGA) deposited exome and RNA-Seq data [24] were used to obtain a complete pan-cancer overview of the genomic and transcriptomic alterations for all *PRDM* genes across 31 distinct human cancer types. 

## 2. Results

### 2.1. Mutational Profiling of PRDM Genes Across Human Cancers

To systematically identify somatic mutations within genes encoding PRDMs, we started with a mutational profiling of these genes across human cancers. We downloaded Exome-sequencing datasets from the TCGA web portal for 31 cancer types and about 11,000 patients. The number of samples for each cancer type is illustrated in Table S1 [25].

**Figure 1:**
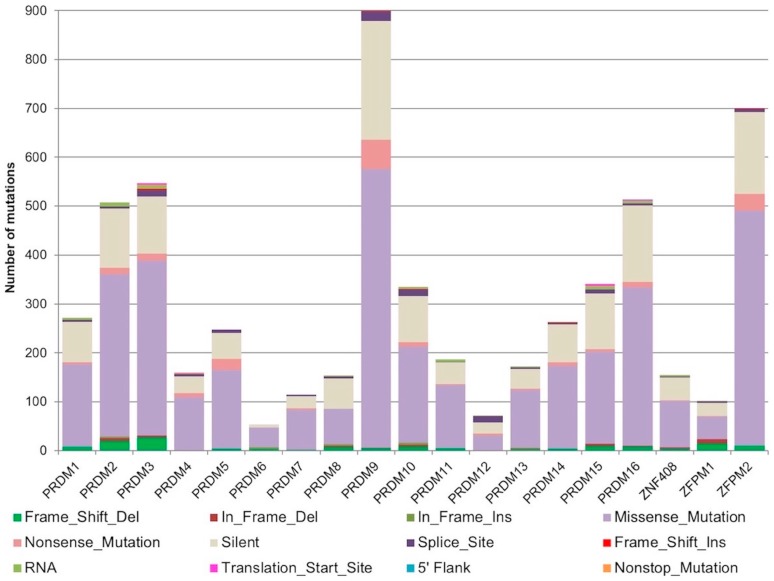
Stacked histograms showing the number of different classes of somatic mutations affecting *PRDM* genes as reported in the Mutation Annotation Files across all analyzed cancer entities.

Overall, we identified 3995 point mutations, 180 deletions (39 in-frame and 141 frameshift), and 22 insertions (16 in-frame and 6 frameshift) affecting PRDM genes. Silent or synonymous mutations were 1531 (26.7% of total mutations) and ranged between 11% (*PRDM6*) and 41% (*PRDM8*) of the total mutations for each gene (Figure 1).

According to our reanalysis, the most mutated genes were *PRDM2* (507 mutations; with 24% of silent mutations), *PRDM3/MECOM* (547 mutations; 22% silent), *PRDM9* (899 mutations; 27% silent), *PRDM16* (514 mutations; 31% silent), and *ZFPM2/FOG2* (700 mutations; 24% silent). Non-sense mutations were more recurrent in *PRDM5* (23), *PRDM9* (60), and *ZFPM2/FOG2* (34), whereas splice sites disrupting mutations were more frequently detected in *PRDM3/MECOM* (13), *PRDM9* (19), *PRDM10* (14) and *PRDM12* (13) (Figure 1).

To measure the frequencies of somatic mutations for each *PRDM* gene across all tumor types, only non-synonymous mutations were considered. We observed heterogeneity in the mutation frequencies of these genes in the different tumor types. A global low mutation rate (from 0 to 8.2%) was found, except for *PRDM3/MECOM*, *PRDM8*, *PRDM9*, *PRDM15*, *ZFPM1/FOG1*, and *ZFPM2/FOG2* (Table 1). In detail, *PRDM8* and *PRDM15* were mutated at low rates in most of the analyzed cancer types except PAAD where they were both frequently mutated (16.0% and 11.2%, respectively). *PRDM3/MECOM* was recurrently mutated in various cancer types, also reaching a value of 20.1% of mutated samples in SKCM. Similarly, *PRDM9* was mutated with a high mutation rate in many cancer types, achieving values of 10.0% in UCEC, 14.2% in LUAD, and 15.4% in SKCM. Otherwise, *ZFPM1/FOG1* was mutated at a low rate in a few cancer types, except in UCS (5.2%), COAD (6.6%), READ (9.4%), and ACC (50.5%). Finally, *ZFPM2/FOG2* was frequently mutated at a high rate in various cancer types, reaching a value of 11.1% in LUAD and 16.5% in SKCM.

We visualized the mutation data in each tumor type by Oncostrip function (Supplementary file 1). Through this approach, we evaluated the percentage of samples with at least one mutated *PRDM* gene in each tumor type ranging from 1.02% (2/196) in LAML samples to 55.43% (51/92) in ACC samples. Furthermore, this function allowed us to visualize the mutation type affecting *PRDM* genes in each sample. Interestingly, *ZFPM1/FOG1* revealed a high number of samples, especially in ACC, with “multi_hit” mutations (more than one mutation affecting the same gene in the same cancer sample). Specifically, we found 11/18 (61%) multi-hit mutations in COAD, 10/11 (90%) in READ, and 23/47 (48%) in ACC (see Supplementary file S1).

To distinguish between damaging and tolerated missense mutations, we carried out a variant effect predictor (VEP) analysis (Table S2). Missense mutations with a SIFT score ranging in the interval 0.0–0.05 and/or with a PolyPhen score in the interval 0.5–1 were considered as deleterious or probably damaging, respectively. As shown in Table 2, adding all the other deleterious somatic mutations (frameshift, in-frame deletions, stop gained and start lost mutations, splice site, UTR, and intron variants) to the deleterious missense mutations classified with the VEP analysis, we obtained the total number of deleterious mutations affecting each *PRDM* gene. Thus, we obtained the percentage of deleterious somatic mutations across the tumor samples. This number was ≥50% for *MECOM/PRDM3* (52.7%), *PRDM4* (55%), *PRDM5* (54.8%), *PRDM6* (58.5%), *PRDM10* (55.2%), *PRDM11* (54%), *PRDM13* (51.7%), and *PRDM16* (50%).

**Figure 2:**
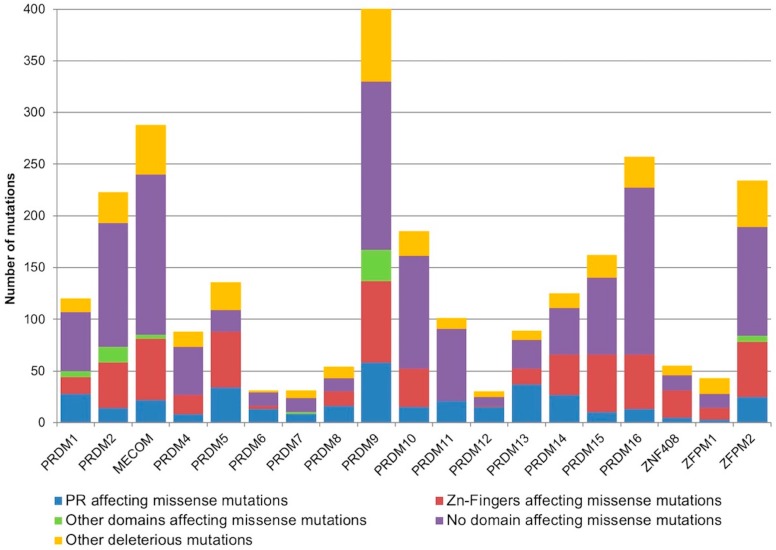
The stacked histogram represents the amount of deleterious somatic mutations affecting the different known domains of PRDM proteins. In detail, the missense mutations affecting the PR domain are reported in blue, the missense mutations affecting the Zn fingers are in red, and the missense mutations affecting other known domains (where present) are illustrated in green. The deleterious missense mutations not affecting known domains are shown in violet and the other classes of deleterious mutations (frameshift, in-frame deletions, stop gained and start lost mutations, splice site, UTR, and intron variants) are in orange.

Additionally, to predict the potential functional effect of the identified *PRDM* somatic mutations on the affected proteins and to detect a possible mutation enrichment in some domains, we localized the deleterious missense mutations on the canonical protein isoform of each *PRDM* (Figure 2). Interestingly, a random sampling weighted on the size of the annotated protein domains demonstrated that somatic deleterious mutations were significantly enriched in the PR domain of *PRDM1*, *PRDM5*, *PRDM6*, *PRDM8*, *PRDM9*, *PRDM12* and *PRDM13* (*p* < 0.005).

**Figure 3:**
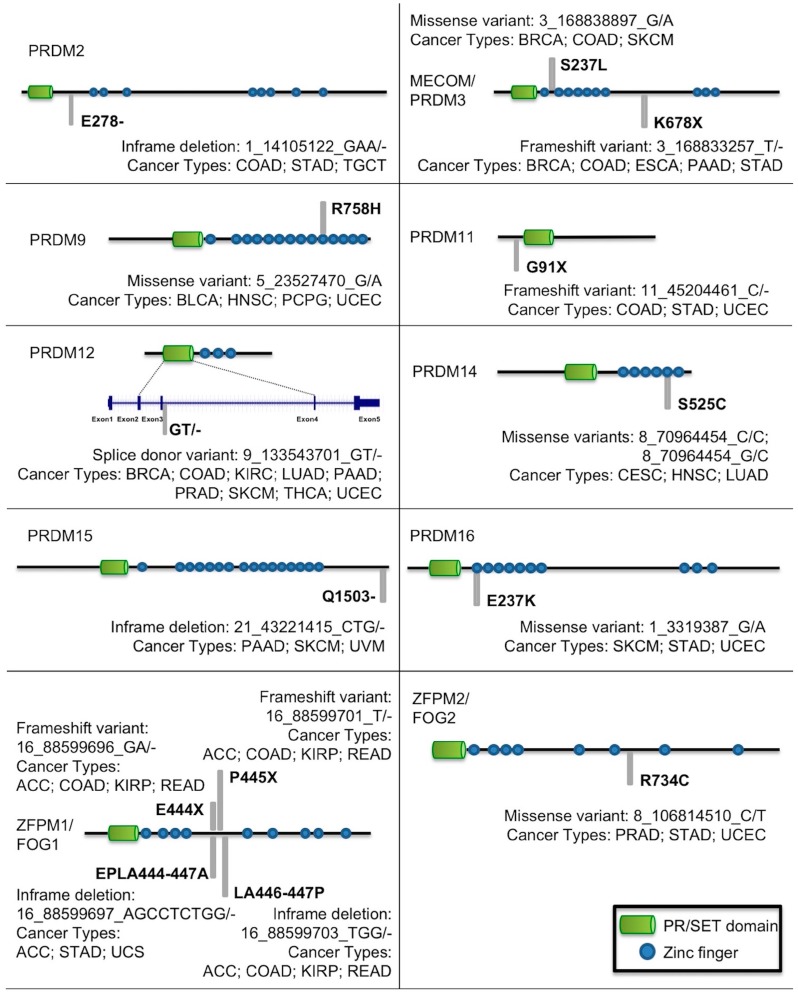
Mutations in *PRDM* genes recurrent in more than three tumor types.

Another important aspect of cancer genetic studies is the presence of possible recurrent and hotspot mutations. Figure 3 illustrates mutations in *PRDM* genes recurring in more than three tumor types. Interestingly, the frameshift mutation T/-→K678X, despite affecting *PRDM3/MECOM* in a region not containing known domains, was recurrent in different tumor types; similarly, also the missense mutation G/A→S237L occurred in a region without known domains but in many tumors. Otherwise, missense mutations affecting a Zn-finger domain and occurring in different tumors were observed for *PRDM9*, *PRDM14*, and *PRDM16*. Likewise, *PRDM12* was frequently mutated in a splice donor site in a region coding for the PR domain whereas in different tumor types, *ZFPM2/FOG2* was affected by the missense mutation C/T→R734C in a region without known domains. In addition, *PRDM2* and *PRDM15* revealed an in-frame deletion in various cancers and *PRDM11* a frameshift mutation. Finally, *ZFPM1/FOG1* showed several recurrent mutations; they all (frameshift mutations and in-frame deletions) hit a region without known domains (Figure 3). 

**Figure 4:**
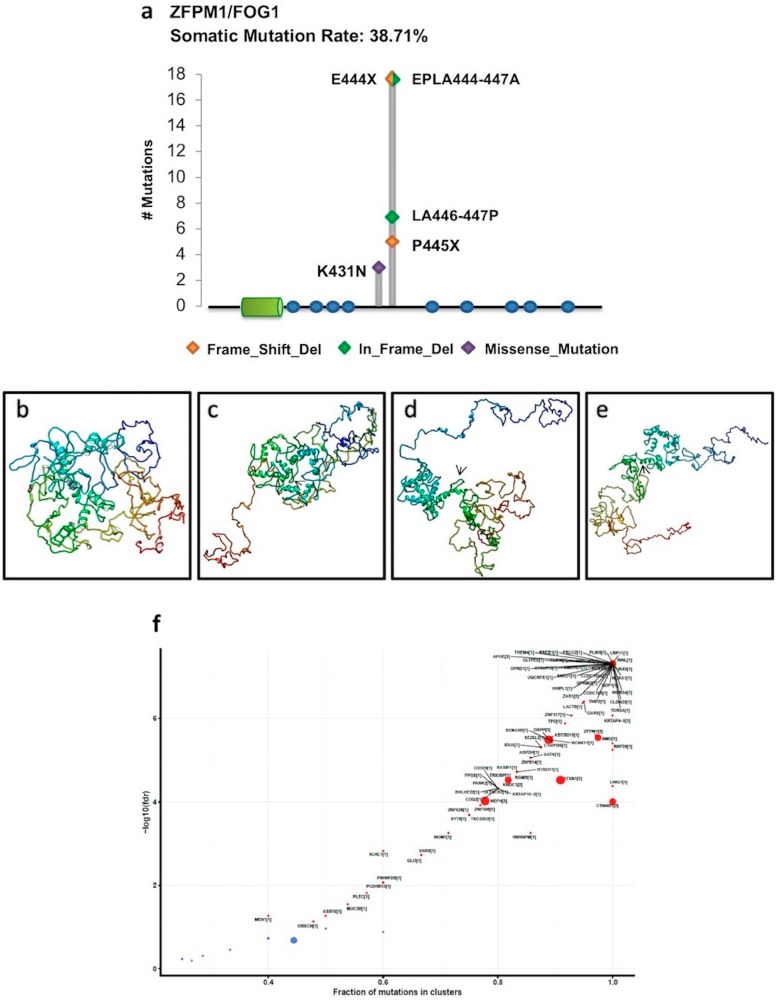
(**a**) *ZFPM1/FOG1* hotspot mutations in ACC obtained by Lollipop plot visualization function. These hotspot mutations are all localized outside the PR domain (green cylinder) and Zinc fingers (blue dots) (**b**–**e**) I-TASSER predicted tertiary structures of the annotated ZFPM1/FOG1 canonical protein (**b**) and ZFPM1/FOG1 proteins carrying the mutations ELPA444-447A (**c**), LA446-447P (**d**), and K431N (**e**); the arrows show the mutated protein regions. (**f**) The scatter plot shows the results of the OncodriveCLUST algorithm analysis for ACC. The dimension of the dots is proportional to the number of clusters found in a certain gene, also indicated in the squared bracket. Specifically, in the *ZFPM1/FOG1* locus, two mutation clusters were found (fdr < 2.87 × 10^−6^).

Interestingly, all these mutations were particularly recurrent in ACC patients. In this cohort, *ZFPM1/FOG1* also displayed five hotspot mutations, all localized in the same region outside the known domains (Figure 4a). To establish whether these hotspot mutations could have an impact on the ZFPM1/FOG1 structure, we utilized the I-TASSER web-tool to predict the tertiary structure of the annotated ZFPM1/FOG1 protein (Figure 4b) and proteins carrying the missense mutations and the in-frame deletions (Figure 4c–e). As illustrated, these mutations completely altered the structure of the canonical protein. Otherwise, the frameshift mutations E444X and P445X led to premature stop codons at the residues 669 and 796, respectively; both of the mutated proteins shared only the first 443 residues with the canonical protein whereas they changed in the 444–669 and 444–796 regions and missed respectively 337 and 210 residues at the C-terminal, which contains the last five zinc fingers of ZFPM1/FOG1.

Finally, to assess whether members of the PRDM family may be driver genes in a given cancer type, we used the OncodriveCLUST tool, which aims to identify genes whose mutations are biased towards a large spatial clustering. This method is based on the feature that cancer gene mutations frequently cluster in particular positions of the protein. Thus, mutations with a frequency higher than the background rate that tend to cluster in specific regions of protein-coding genes are likely to be driver genes. Based on the scores of this analysis, *ZFPM1/FOG1* can be considered as a cancer driver for ACC (Figure 4f) and *PRDM8* for PAAD (Figure S2).

### 2.2. Differentially Expressed PRDM Genes across Human Cancers

**Figure 5:**
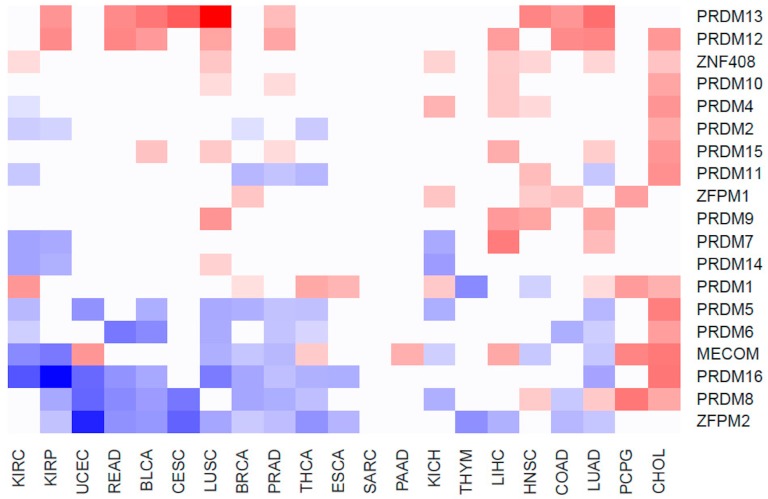
The heatmap shows the expression profiles of *PRDM*s across the analyzed cancer types.

To evaluate whether the expression of *PRDM* genes is affected in human cancers, we took advantage of RNA-Seq datasets from paired samples (cancer vs. benign counterpart) available at the TCGA web portal. Globally, 585 patients across 21 cancer types were analyzed (Table S1). The gene expression profiles differed considerably between normal and tumor specimens, depending on the cancer type, as shown by the principal component analysis [26]. The results of gene expression profiling are summarized in Table S3 and Figure 5.

Data indicate that a large subset of *PRDM* genes is consistently deregulated across several cancer types. Particularly, a significant overexpression was measured for *PRDM12* and *PRDM13*. On the other side, *ZFPM2/FOG2*, *PRDM8*, and *PRDM16* were more often downregulated across tumors (Figure 5). Strong upregulation of almost all *PRDM* genes was measured in CHOL where 13/19 PRDM genes were overexpressed in tumor versus healthy counterparts. Among them, the most upregulated were *MECOM/PRDM3* (FC = 12.64), *PRDM5* (FC = 10.7) and *PRDM16* (FC = 13.74). Conversely, the cancer types with the smallest number of deregulated *PRDM* genes were SARC with no *PRDM* genes deregulated, PAAD with 1 gene upregulated (*MECOM/PRDM3*, FC = 2.71), THYM with two genes strongly downregulated (*PRDM1*, FC = 0.13 and *ZFPM2/FOG2*, FC = 0.16), CESC with two genes strongly downregulated (*PRDM8*, FC = 0.07 and *ZFPM2/FOG2*, FC = 0.04) and one gene strongly upregulated (*PRDM13* FC = 29.86), and, finally, ESCA with two genes strongly downregulated (*PRDM16*, FC = 0.37 and *ZFPM2/FOG2*, FC = 0.44) and one gene upregulated (*PRDM1*, FC = 2.28).

### 2.3. PRDM Expression in Human Primary Tumors

In an attempt to validate the findings obtained on RNA-Seq datasets, we assayed TissueScan cDNA panel arrays containing eight different tumors (breast, colon, kidney, liver, lung, ovary, prostate, and thyroid) [25]. 

**Figure 6:**
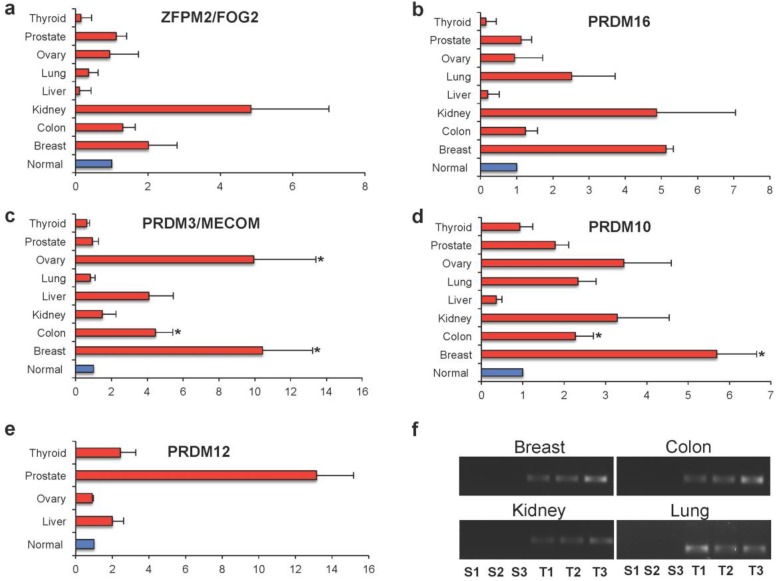
Relative expressions obtained by real-time PCR in different types of cancer tissues versus corresponding normal tissues (with arbitrary expression value equal to 1). The comparative threshold cycle (Ct) method was used with β-actin as the internal control. The results are expressed as the mean ± ES. The statistical significance of differences between experimental groups was calculated using the unpaired two-tailed Student’s *t*-test. (*) Results with a *p*-value < 0.05 were considered significant. (**a**) *ZFPM2/FOG2*; (**b**) *PRDM16*; (**c**) *PRDM3/MECOM*; (**d**) *PRDM10*; (**e**) *PRDM12*. (**f**) Representative samples analyzed for *PRDM12* by agarose gel electrophoresis.

Specifically, we analyzed the differential expression of *PRDM3/MECOM*, *PRDM10*, *PRDM12*, *PRDM16*, and *ZFPM2/FOG2* genes (Figure S3). As illustrated in Figure 6, a general differential expression, even though not significant, was observed for both *ZFPM2/FOG2* and *PRDM16* in several tumor tissues (Figure 6a,b). *PRDM3/MECOM* was found to be significantly overexpressed in breast (*p* < 0.001), ovary (*p* < 0.001), and colon (*p* < 0.05) cancer specimens (Figure 6c). Similarly, *PRDM10* was overexpressed at significant levels in breast (*p* < 0.001) and colon (*p* < 0.05) cancer specimens (Figure 6d). No statistical differences in the expression of all these genes were measured between tumor and healthy samples of the other cancer entities (Figure 6a–d). Otherwise, the *PRDM12* gene was difficult to analyze. This gene was confirmed as being highly overexpressed in all the analyzed cancer tissues, except in ovarian cancer, as indicated by reanalysis of the TCGA RNA-Seq dataset. However, this gene was not expressed or was expressed at very low levels in all the healthy tissues used as controls; it was expressed in tumor specimens. For this reason, its relative gene expression could not be calculated using the 2^−ΔΔ*C*t^ method for all the analyzed tissues. Particularly, relative expression was measured only for thyroid, ovary, prostate, and liver (Figure 6e) whereas in breast, colon, kidney, and lung normal tissues, the amplification products were undetectable when observed by agarose gel electrophoresis analysis (Figure 6f). Of note, these cancer specimens showed measurable levels of *PRDM12* (Figure 6e–f).

## 3. Discussion

In this study, we provide for the first time a systematic and comprehensive overview of both the mutational status and the expression profile of all the *PRDM* genes across a large number of different cancer types.

Recently, the availability of multi-omics datasets (such as TCGA) from human cancers, together with the development of advanced bioinformatics tools, represent a unique source to study human malignancies [24].

Our reanalysis of the TCGA Exome-sequencing datasets revealed *PRDM2*, *PRDM3/MECOM*, *PRDM9*, *PRDM16* and *ZFPM2/FOG2* as the most mutated genes. Heterogeneity in the mutation frequencies was observed in the different tumor types with higher mutation rates found for *PRDM3/MECOM*, *PRDM8*, *PRDM9*, *PRDM15*, *ZFPM1/FOG1* and *ZFPM2/FOG2* in specific cancers. Remarkably, VEP analysis indicated that the percentage of total deleterious mutations across the tumor samples was high for most genes. More interestingly, a random sampling weighted on the size of the annotated protein domains demonstrated that deleterious mutations were significantly enriched in the PR domain of *PRDM1*, *PRDM5*, *PRDM6*, *PRDM8*, *PRDM9*, *PRDM12* and *PRDM13*. Frequent mutations disrupting the PR domain in tumor samples would be a mechanism for removing the tumor suppressor function of the PR-plus isoform in favor of the oncogenic PR-minus form.

A big challenge in cancer biology studies is distinguishing between mutations conferring a selective growth advantage to cancer cells (drivers) and those randomly accumulating and without significant effects on the oncogenic process (passengers) [27,28]. Many algorithms employing different approaches are now utilized to recognize driver genes although, when compared for their performance, all display both strengths and weaknesses [25,29,30,31]. Moreover, recent studies have highlighted the existence of “mini-driver” genes with weaker tumor-promoting effects, thus expanding the previous driver–passenger dichotomy to a continuous model [25,30,32]. Besides, a sub-classification has been proposed to differentiate “mut-driver genes”, usually altered by somatic gene mutations, from “epi-driver genes”, which are deregulated through epigenetic modifications but are not frequently mutated [25,27].

In this study, OncodriveCLUST analysis revealed two putative cancer mut-driver genes: *PRDM8* in PAAD and *ZFPM1/FOG1* in ACC. In the latter case, we found that the involved gene was mutated in a very high percentage (about 55%) of tumor samples. Additionally, a mutational hotspot region localized between the amino acid positions 444 and 447, outside the known domains, was recognizable. All these findings agree with the key parameters commonly used to discern drivers from passengers [29]. Notably, these hotspot mutations recurred also in other malignancies, such as COAD, KIRP, READ, STAD, and UCS, supporting an important role of this gene in carcinogenesis. Moreover, the finding of “multi_hit” mutations in ACC, as well as in COAD and READ, advises that this gene could function as a tumor suppressor gene. This is conceivable with the current knowledge about the role of *ZFPM1/FOG1* in differentiation. Indeed, ZFPM1 is also known as a friend of GATA1 (FOG1) since it interacts with GATA1 and it is an essential cofactor for the transcription factor GATA-1 in erythroid and megakaryocytic differentiation. Reduced expression of ZFPM1/FOG1 was found in preleukemic progenitors of a mouse model of leukemia [33,34]. Besides, the high recurrence of these mutations together with results from 3D-modeling of the canonical and mutant *ZFPM1/FOG1* proteins suggest that, although without known domains, this is a critical region for the protein. Interestingly, this region (particularly K431 residue) is evolutionary highly conserved (Figure S4).

It is conceivable that other *PRDM* genes may play a key role in the initiation and progression of specific or multiple tumor types, as also reported by previous literature [8,15,16,17]. Indeed, it is accepted that cancer driver genes are mainly involved in three core cellular processes: cell fate, cell survival, and genome maintenance. For instance, *PRDM2* has a relevant role in all of them; specifically, it also participates to the formation of protein complexes involved in the DNA damage response and in genome maintenance [15]. Additionally, a recent study identified a driver *PRDM2* mutation in MSI colorectal cancer [16]. However, our analysis has not considered this gene as a driver. We cannot exclude, among the explanations, the limitation of the utilized bioinformatics tool [30].

Our pan-cancer study has also identified *PRDM12* as a possible epi-driver gene in multiple cancers. Of note, TCGA gene expression profiling of PRDM genes revealed significant overexpression of *PRDM12* and *PRDM13* in many tumor types whereas *ZFPM2/FOG2*, *PRDM8*, and *PRDM16* were more often downregulated in tumor tissues. Our qRT-PCR analysis was not able to confirm all the results obtained through TCGA analysis. The main reason could be the utilization of unpaired samples in the validation through TissueScan cDNA panel arrays; otherwise, our analysis on TCGA was carried out on paired samples. In addition, we have analyzed a small number of samples by qRT-PCR compared to the huge number of cases from TCGA. Noteworthy is that when we measured the differential expression of *PRDM12* in cDNA panel arrays, we found the expression of this gene only in cancer specimens but not in healthy samples of several tissues, suggesting that it could be putatively utilized as a biomarker in those malignancies. Our study represents the first analysis of all *PRDM* genes in pan-cancer; further studies using large cohorts are necessary to validate the most promising results, particularly for *PRDM12*. In addition, given the lack of literature data, we are aware that functional studies investigating the effect of altered expression both in vitro and in vivo are required to establish the possible impact of *PRDM12* in cancer. 

Altogether, our results can be useful for identifying a subset of relevant *PRDMs* that are frequently mutated and/or transcriptionally deregulated in certain tumor types. Functional studies on specific *PRDM* gene mutations should be accomplished to definitely prove their potential oncogenic role. Moreover, it would be interesting to investigate whether these mutations contribute to cancer progression and metastasis, as well as whether they correlate with prognosis and/or with drug response and resistance. The epigenetic changes underlying the altered gene expression observed in tumor samples should also be explored. In this context, the availability of novel multi-omics data integration tools and methods also offer the opportunity to further integrate our analysis of *PRDM* gene expression by a systematic pan-cancer study of the epigenetic marks in these genes [25,35,36].

## 4. Materials and Methods

### 4.1. TCGA Data Source Selection and Processing for Mutation Analysis

In this manuscript, we analyzed both whole Exome- and RNA-Seq data retrieved from publicly accessible repositories. Specifically, we retrieved the whole exome sequencing data from the GitHub R data package for pre-compiled somatic mutations from TCGA cohorts “TCGA mutations” and analyzed it using the Bioconductor package “maftools” [37]. 

The selection and nomenclature of *PRDM* genes were based on the HUGO Gene Nomenclature Committee [38]. To estimate the mutation enrichment within the PR domain of each of the PRDM proteins, we performed a random sampling iterated 1000 times weighted on the size of the annotated domains.

To assess whether one or more PRDM proteins could be considered as cancer driver genes based on the positional clustering of the variants in the selected human cancers, we used a re-implementation of the software OncodriveCLUST within the maftools package [39].

Three-dimensional (3D) modeling of the human canonical and mutant ZFPM1/FOG1 proteins was carried out using I-TASSER [26,40,41].

### 4.2. TCGA Data Source Selection and Processing for Expression Analysis

The RNA-Seq gene expression data were downloaded from TCGA [42]. The analysis of gene expression and the identification of differentially expressed genes were performed comparing the expression profiles of cancer vs. matched normal samples in a paired analysis. Therefore, expression data taken from human primary cancers for which healthy samples were not available were discarded. According to this criterion, 22 tumor entities were analyzed. To have a more robust differential expression analysis in paired samples, we applied generalized linear models implemented in the EdgeR Bioconductor package version 3.17.10. *p*-values adjustment was performed through the application of the false discovery rate (FDR) method. We considered differentially expressed genes with a logFC ≤−1 and logFC ≥1, and an FDR ≤ 0.01.

### 4.3. Real-Time RT-PCR Analysis

Quantitative real-time PCR (qRT-PCR) experiments were carried out on TissueScan Cancer Survey Panels, which contained cDNA samples from various normal and cancer tissues covering eight different tumors (breast, colon, kidney, liver, lung, ovary, prostate and thyroid) from independent patients diagnosed at various clinical disease stages and selected from mixed ages and genders. Tissue cDNAs of each array were synthesized from high-quality total RNAs of pathologist-verified tissues, normalized and validated with β-actin in two sequential qPCR analyses, and provided with clinical information and QC data [25].

To quantitatively determine the relative amount of *PRDM3/MECOM*, *PRDM8*, *PRDM10*, *PRDM12*, *PRDM16*, and *ZFPM2/FOG2* RNAs, qRT-PCR was performed [25]. Primers were designed using Primer3Plus [43] and specificity was verified with the BLAST program and through *in-silico* PCR analysis by the UCSC Genome Browser [44].

The selected sequences of oligonucleotides forward (F) and reverse (R) were: *PRDM3/MECOM*-F 5′-AGTGGCAGTGACCTGGAAAC-3′; *PRDM3/MECOM*-R 5′-ACCGCAGTCTGCTCCTCTAA-3′; *PRDM10*-F 5′-CAGCACATTCGAAAGAAGCA-3′; *PRDM10*-R 5′-GCGTTCGGTAGTCTGTCGTT-3′; *PRDM12*-F 5′-GGGAGTCCTTACGCAACCTT-3′; *PRDM12*-R 5′-TTCCATTGTGCCTCCACTCT-3′; *PRDM16*-F 5′-ATGATGGACAAGGCAAAACC-3′; *PRDM16*-R 5′-GATGTGGGAGGTAGCAGAGG-3′; *ZFPM2/FOG2*-F 5′-GACAGTGCCCATCAGATTTC-3′; *ZFPM2/FOG2*-R 5′-GGGCAGGAATTCTTC CATTTT-3′.

The amplification products were also analyzed by agarose gel electrophoresis [45]. Data were normalized with β-actin gene provided with arrays. The relative gene expression was calculated using the 2^−ΔΔ*C*t^ method [25].

## Figures and Tables

**Table 1:** Frequency of patients carrying mutations in the *PRDMs* across the 31 analyzed tumors.

Cancers	*Genes*																		
	***PRDM1***	***PRDM2***	***MECOM***	***PRDM4***	***PRDM5***	***PRDM6***	***PRDM7***	***PRDM8***	***PRDM9***	***PRDM10***	***PRDM11***	***PRDM12***	***PRDM13***	***PRDM14***	***PRDM15***	***PRDM16***	***ZNF408***	***ZFPM1***	***ZFPM2***
**ACC**	0	1.1	0	1.1	1.1	2.2	0	0	3.2	1.1	0	0	1.1	0	0	1.1	0	**50.5**	4.3
**BLCA**	1.7	4.1	**5.3**	3.4	3.4	0	1.9	1	3.9	2.2	1.7	0	1.4	1.7	2.7	4.1	0.7	1	3.6
**BRCA**	0.8	0.9	1	0.5	0.3	0.2	0.2	0.5	0.7	0.8	0.2	0.2	0.3	0.5	0.6	0.5	0.1	0.2	1.3
**CESC**	0.5	4	2	1.5	2	0.5	1	0.5	4.5	1	1.5	0	0	0.5	3	3	1	0.5	1
**CHOL**	2.7	0	0	**5.4**	0	2.7	0	0	**5.4**	0	0	0	0	0	0	0	2.7	0	0
**COAD**	2.2	**6.6**	**5.5**	1.5	2.6	2.9	0.7	1.1	**5.5**	4.4	1.1	0.4	1.5	1.8	2.2	4.4	1.5	**6.6**	2.6
**DLBC**	**8.2**	2	2	0	2	0	0	2	**8.2**	0	0	0	0	0	2	**6.1**	0	0	4.1
**ESCA**	1.1	1.6	3.2	2.1	1.6	2.7	1.1	1.6	**7**	4.8	1.6	0	2.1	2.7	4.8	2.7	1.6	0.5	**8.6**
**GBM**	0.6	0.6	2.8	0.3	1.1	0	0.3	0	3.3	1.9	0.6	0.8	0.3	0.3	1.9	0.6	0.6	0	0.3
**HNSC**	1.3	2.1	2.5	0.8	1.3	0	0.6	0.6	**7.2**	1.1	1.1	0.8	0.8	1.9	0.9	2.3	0.6	0.9	0.8
**KICH**	0	1.5	1.5	0	0	0	0	1.5	**6**	0	0	1.5	1.5	0	0	1.5	0	0	0
**KIRC**	0.2	0.8	1.2	1.5	0.3	0.2	0.8	0	1	1	0.2	0.3	0.7	0.5	1.2	0.5	0.5	0.2	1.2
**KIRP**	0.3	2.4	0	0.3	0.3	0.7	0.3	0.3	0.7	1	0.7	0.3	1.4	1	1.7	1.4	1.4	0.3	0.3
**LAML**	0	0	0	0	0	0	0	0	0.5	0	0	0	0	0	0	0.5	0	0	0
**LIHC**	1.6	3.2	2.9	1.8	1.1	0.8	0.5	0.8	2.4	1.6	1.8	0	0.8	1.3	2.4	2.1	0.8	0	3.9
**LUAD**	1.9	3.7	2.1	1.6	1.9	0	1.9	1.2	**14.2**	3	1.6	1.1	0.9	4.4	2.3	4.2	0.9	0.2	**11.1**
**LUSC**	2.8	3.4	**5**	0	2.8	0	0.6	0	**7.3**	3.4	2.2	1.1	3.4	0	2.2	3.4	1.7	0	5
**OV**	0.2	0.4	0	0	0	0	0.4	0	1.3	0	0.2	0	0	0.2	0.2	0	0	0	1.3
**PAAD**	0.5	4.3	2.7	1.1	1.6	0	2.1	**16**	1.6	3.2	2.7	2.7	1.6	0.5	**11.2**	1.6	1.1	0	2.7
**PCPG**	0	0.5	0	0	0	0	0	0	1.1	0	0	0	0	0	0	0	0.5	0	0.5
**PRAD**	0	0.8	1.4	0	0.6	0	0.6	0.6	0.8	0.4	0.2	0.4	0.2	0.4	0.4	0.8	0.6	0.6	1.4
**READ**	0	2.6	1.7	2.6	3.4	0.9	0	0	2.6	1.7	0.9	0.9	0	0	1.7	0.9	0	**9.4**	**5.1**
**SARC**	1.1	1.9	1.9	1.1	0.8	2.7	0.8	0.4	3	1.5	1.1	0.4	0.8	1.5	3.4	3	0.8	0.8	2.3
**SKCM**	**5.3**	4.2	**20.1**	1.3	3.6	0	2.3	1.5	**15.4**	3.6	3	1.3	2.5	3.6	3.6	**7.8**	1.9	0.8	**16.5**
**STAD**	2.3	**7.8**	3.8	1	3	0	1	2.3	**5.8**	4.3	1.5	0.3	2.8	2.5	3	4.8	1.8	1	**5.8**
**TGCT**	0	2.5	0.6	0	0	1.3	0.6	1.9	2.5	2.5	0.6	0	1.3	0	0	1.3	0	1.3	0
**THCA**	0.4	0.6	0	0.4	0.2	0	0.8	0.4	1.2	0.8	0.2	0.2	0.2	0	0.4	0.8	0.4	0.4	0.4
**THYM**	2.4	3.2	3.2	2.4	0	2.4	3.2	0.8	3.2	1.6	0.8	0.8	0.8	2.4	2.4	3.2	0.8	0.8	1.6
**UCEC**	4	**7.2**	**5.6**	2.4	4.4	2	0.8	1.6	**10**	**5.2**	2.4	1.2	2.8	4.8	3.2	**5.6**	4	0.4	4.8
**UCS**	**5.2**	1.7	0	0	3.4	0	1.7	1.7	1.7	1.7	1.7	0	1.7	0	3.4	0	1.7	**5.2**	**5.2**
**UVM**	0	1.2	0	1.2	1.2	0	0	1.2	4.9	0	1.2	0	0	0	1.2	0	0	0	0

**Table 2:** Percentage of deleterious and tolerated mutations in the *PRDMs* across the analyzed tumor samples.

Genes	Deleterious Mutations	Total Mutations	% Deleterious Mutations
***PRDM1***	120	272	44.1
***PRDM2***	223	507	44.0
***MECOM/PRDM3***	288	547	52.7
***PRDM4***	88	160	55.0
***PRDM5***	136	248	54.8
***PRDM6***	31	53	58.5
***PRDM7***	31	114	27.2
***PRDM8***	54	154	35.1
***PRDM9***	403	899	44.8
***PRDM10***	185	335	55.2
***PRDM11***	101	187	54.0
***PRDM12***	30	72	41.7
***PRDM13***	89	172	51.7
***PRDM14***	125	263	47.5
***PRDM15***	162	341	47.5
***PRDM16***	257	514	50.0
***ZNF408/PRDM17***	55	155	35.5
***ZFPM1/FOG1***	43	102	42.2
***ZFPM2/FOG2***	234	700	33.4

## Supplementary Materials

Supplementary materials can be found at http://www.mdpi.com/1422-0067/19/10/3250/s1.

## Abbreviations

FC	Fold change
FDR	False discovery rate
TCGA	The Cancer Genome Atlas
ACC	Adrenocortical carcinoma
BLCA	Bladder cancer
BRCA	Breast cancer
CESC	Cervical squamous cell carcinoma and endocervical adenocarcinoma
CHOL	Cholangiocarcinoma
COAD	Colon adenocarcinoma
DLBC	Lymphoid neoplasm diffuse large B-cell lymphoma
ESCA	Esophageal carcinoma
GBM	Glioblastoma
HNSC	Head and neck squamous cell carcinoma
KICH	Kidney chromophobe carcinoma
KIRC	Kidney renal clear cell carcinoma
KIRP	Kidney renal papillary cell carcinoma
LAML	Acute myeloid leukemia
LIHC	Liver hepatocarcinoma
LUAD	Lung adenocarcinoma
LUSC	Lung squamous cell carcinoma
OV	Ovarian cancer
PAAD	Pancreas adenocarcinoma
PCPG	Pheochromocytoma and paraganglioma
PRAD	Prostate adenocarcinoma
READ	Rectum adenocarcinoma
SARC	Sarcoma
SKCM	Skin cutaneous melanoma
STAD	Stomach adenocarcinoma
TGCT	Testicular germ cell tumors
THCA	Thyroid cancer
THYM	Thymoma
UCEC	Uterine corpus endometrial carcinoma
UCS	Uterine carcinosarcoma
UVM	Uveal melanoma
