# Supplementary material for: PR/SET Domain Family and Cancer: Novel Insights from The Cancer Genome Atlas
Source: Int J Mol Sci. 2018 Oct 19;19(10):3250. doi: 10.3390/ijms19103250 (PMC6214140; doi:10.3390/ijms19103250)
Supplement: Supplementary file 1 [file ijms-19-03250-s001.zip › SUPPLEMENTARY/Figure S4.pdf]

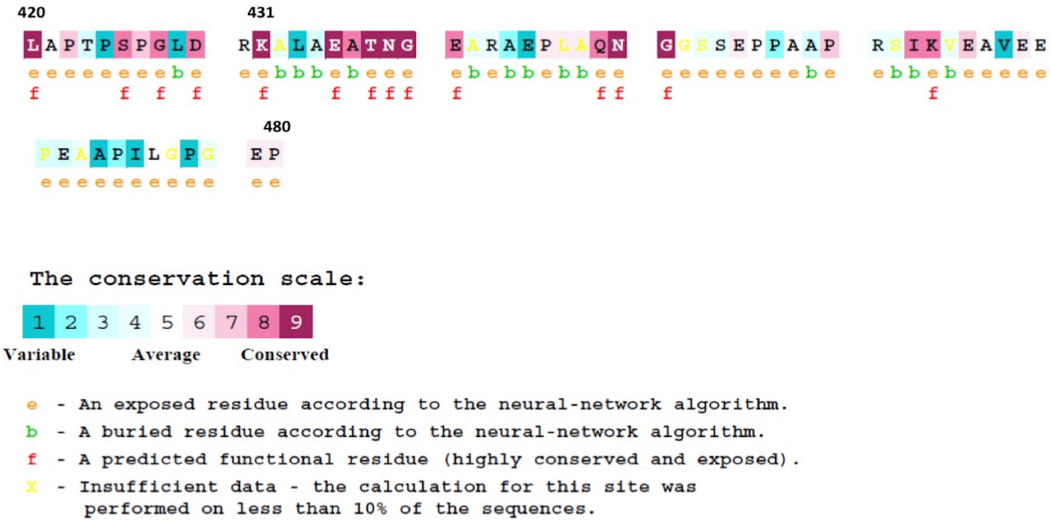

Figure S4. ZFMP1/FOG1 ConSurf conservation analysis. The figure illustrates the conservation results for protein interval L420 – P480 using the color-coding bar; the highly variable and conserved residues are shown in turquoise and maroon, respectively. Yellow colored residues denote regions with low confidence of conservation level due to insufficient data and hence excluded from the analysis. [The ConSurf web server (http://consurf.tau.ac.il)]
