# Supplementary material for: PR/SET Domain Family and Cancer: Novel Insights from The Cancer Genome Atlas
Source: Int J Mol Sci. 2018 Oct 19;19(10):3250. doi: 10.3390/ijms19103250 (PMC6214140; doi:10.3390/ijms19103250)
Supplement: Supplementary file 1 [file ijms-19-03250-s001.zip › SUPPLEMENTARY/Supplementary File_S1.pdf]

SUPPLEMENTARY FILE_S1

Oncostrip plots – The Oncostrip graphs show the distribution of all the mutations in the selected genes across the cohort of mutated patients. The colors indicate the variant classification of the mutations.
Below are the graphs concerning the following tumors in order: ACC, BLCA, BRCA, CESC, CHOL, COAD, DLBC, ESCA, HNSC, KICH, KIRC, KIRP, LAML, LIHC, LUAD, LUSC, OV, PAAD, PCPG, PRAD, READ, SARC, SKCM, STAD, TGCT, THCA, THYM, UCEC, UCS, UVM.

Altered in 51 (55.43%) of 92 samples.

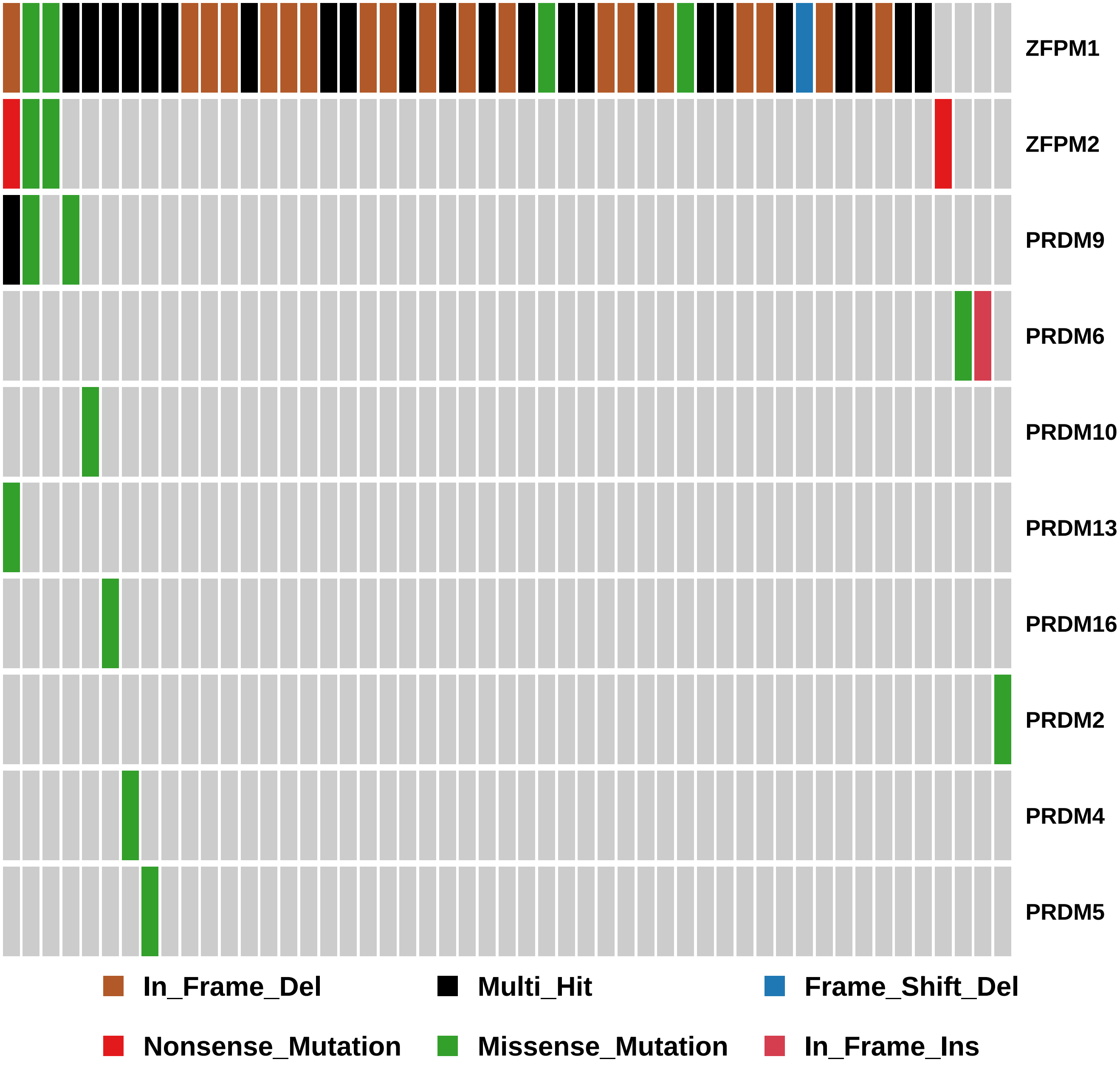

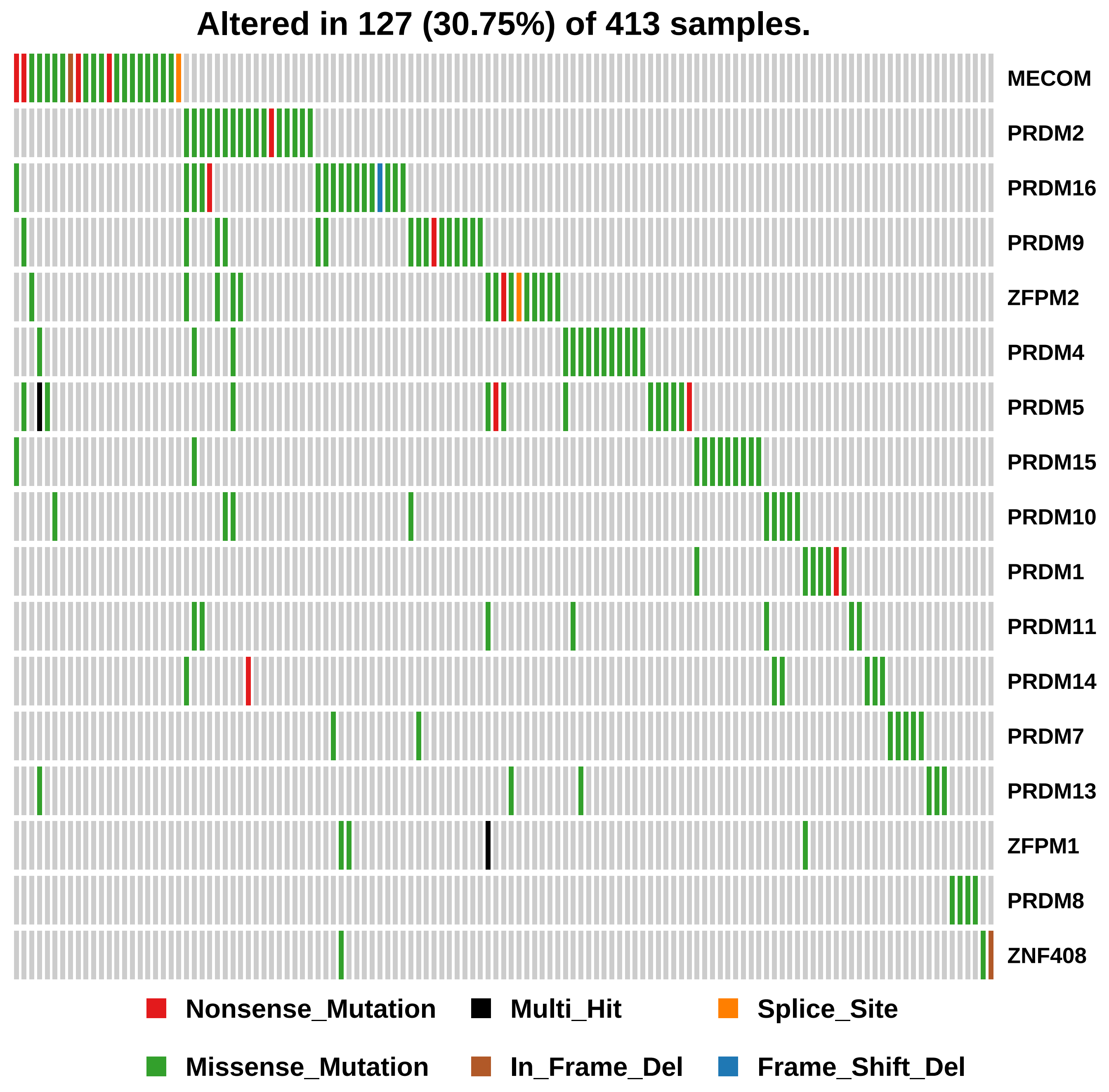
Altered in 127 (30.75%) of 413 samples.
MECOM
PRDM2
PRDM16
PRDM9
ZFPM2
PRDM4
PRDM5
PRDM15
PRDM10
PRDM1
PRDM11
PRDM14
PRDM7
PRDM13
ZFPM1
PRDM8
ZNF408
Nonsense_Mutation
Multi_Hit
Splice_Site
Missense_Mutation
In_Frame_Del
Frame_Shift_Del

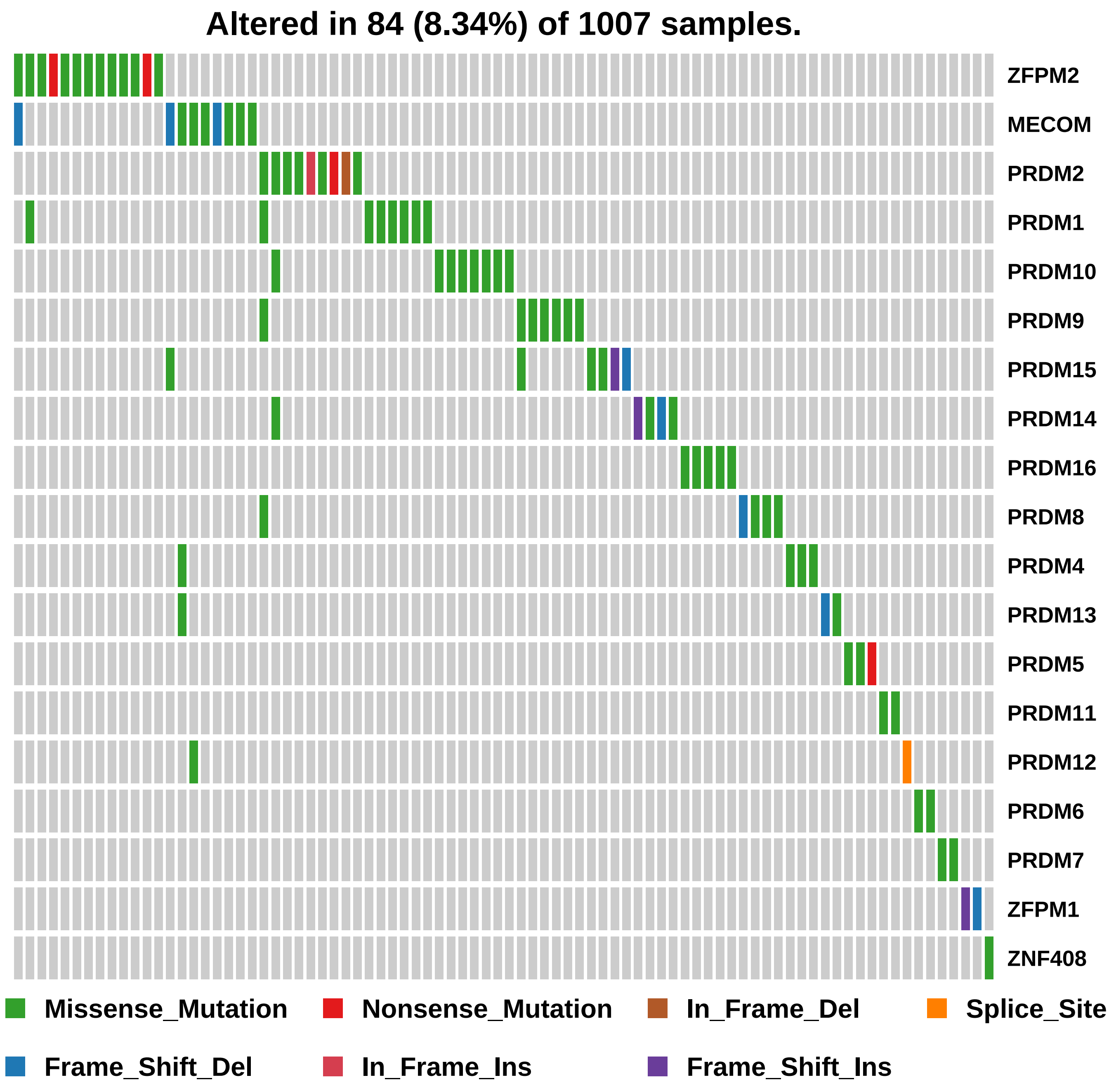
Altered in 84 (8.34%) of 1007 samples.
ZFPM2
MECOM
PRDM2
PRDM1
PRDM10
PRDM9
PRDM15
PRDM14
PRDM16
PRDM8
PRDM4
PRDM13
PRDM5
PRDM11
PRDM12
PRDM6
PRDM7
ZFPM1
ZNF408
Missense_Mutation
Nonsense_Mutation
In_Frame_Del
Splice_Site
Frame_Shift_Del
In_Frame_Ins
Frame_Shift_Ins

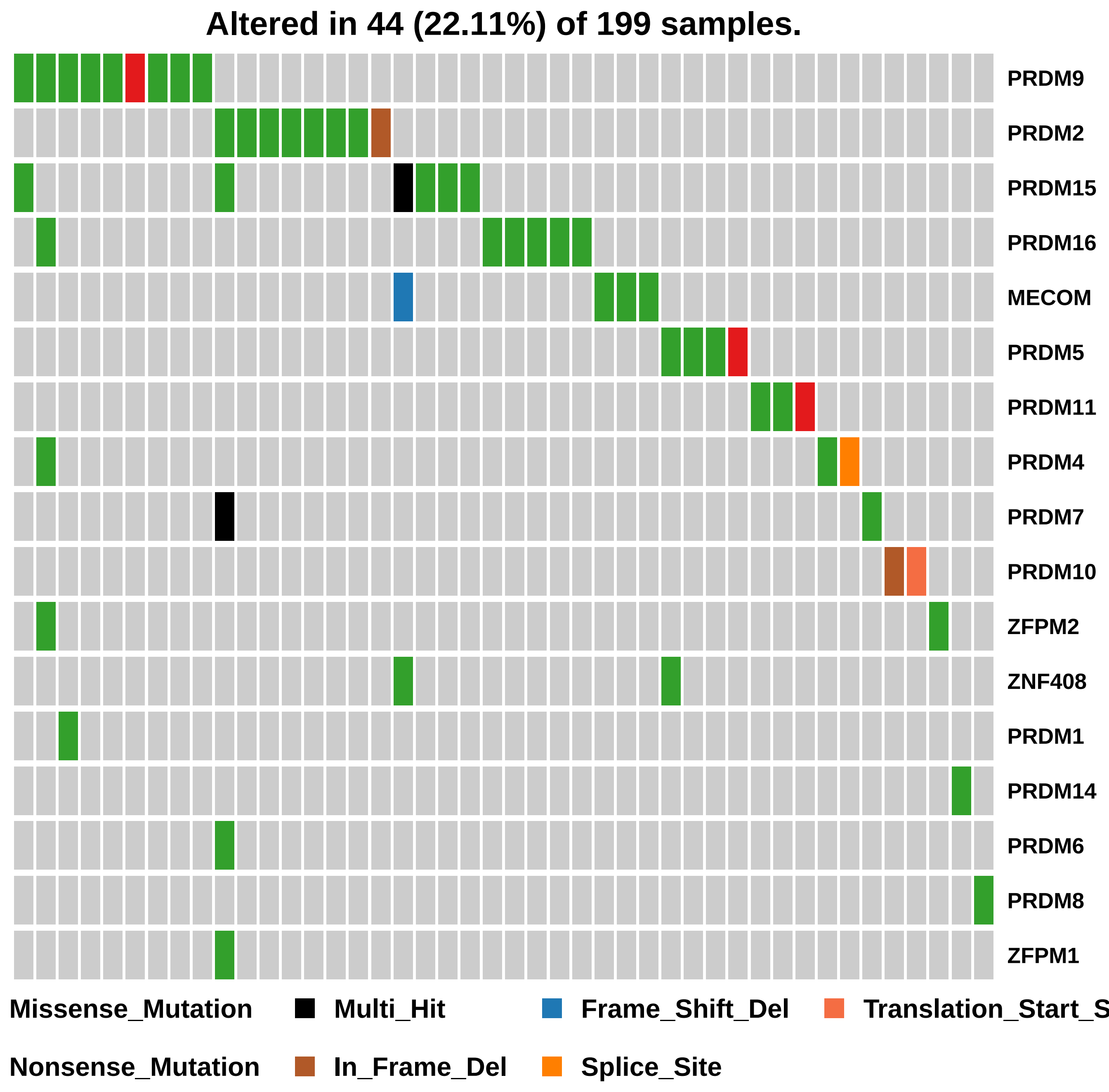

Altered in 44 (22.11%) of 199 samples.
PRDM9
PRDM2
PRDM15
PRDM16
MECOM
PRDM5
PRDM11
PRDM4
PRDM7
PRDM10
ZFPM2
ZNF408
PRDM1
PRDM14
PRDM6
PRDM8
ZFPM1
Missense_Mutation
Multi_Hit
Frame_Shift_Del
Nonsense_Mutation
In_Frame_Del
Splice_Site

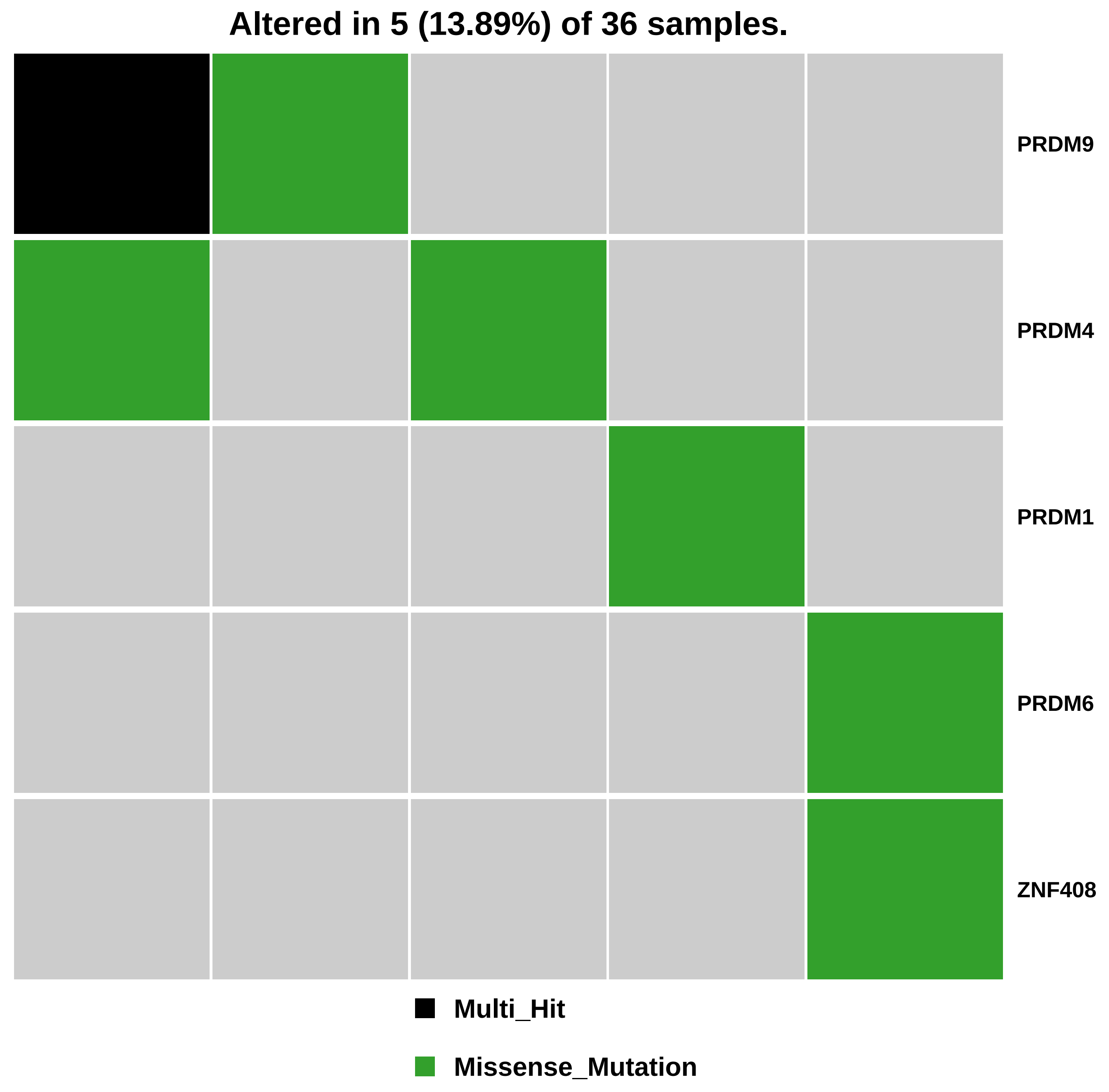
Altered in 5 (13.89%) of 36 samples.
PRDM9
PRDM4
PRDM1
PRDM6
ZNF408
Multi_Hit
Missense_Mutation

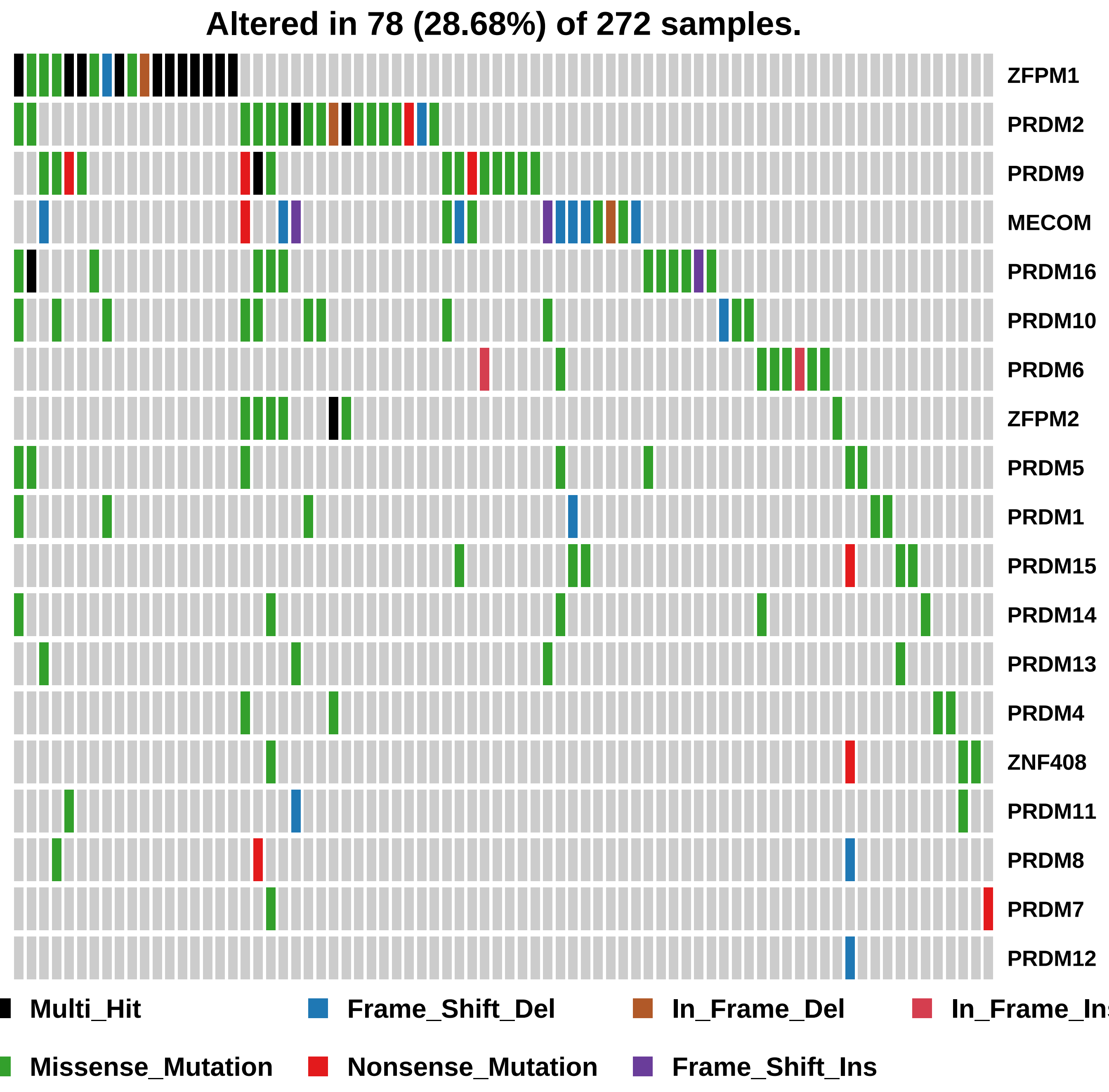
Altered in 78 (28.68%) of 272 samples.
ZFPM1
PRDM2
PRDM9
MECOM
PRDM16
PRDM10
PRDM6
ZFPM2
PRDM5
PRDM1
PRDM15
PRDM14
PRDM13
PRDM4
ZNF408
PRDM11
PRDM8
PRDM7
PRDM12
Multi_Hit
Frame_Shift_Del
In_Frame_Del
Missense_Mutation
Nonsense_Mutation
Frame_Shift_Ins

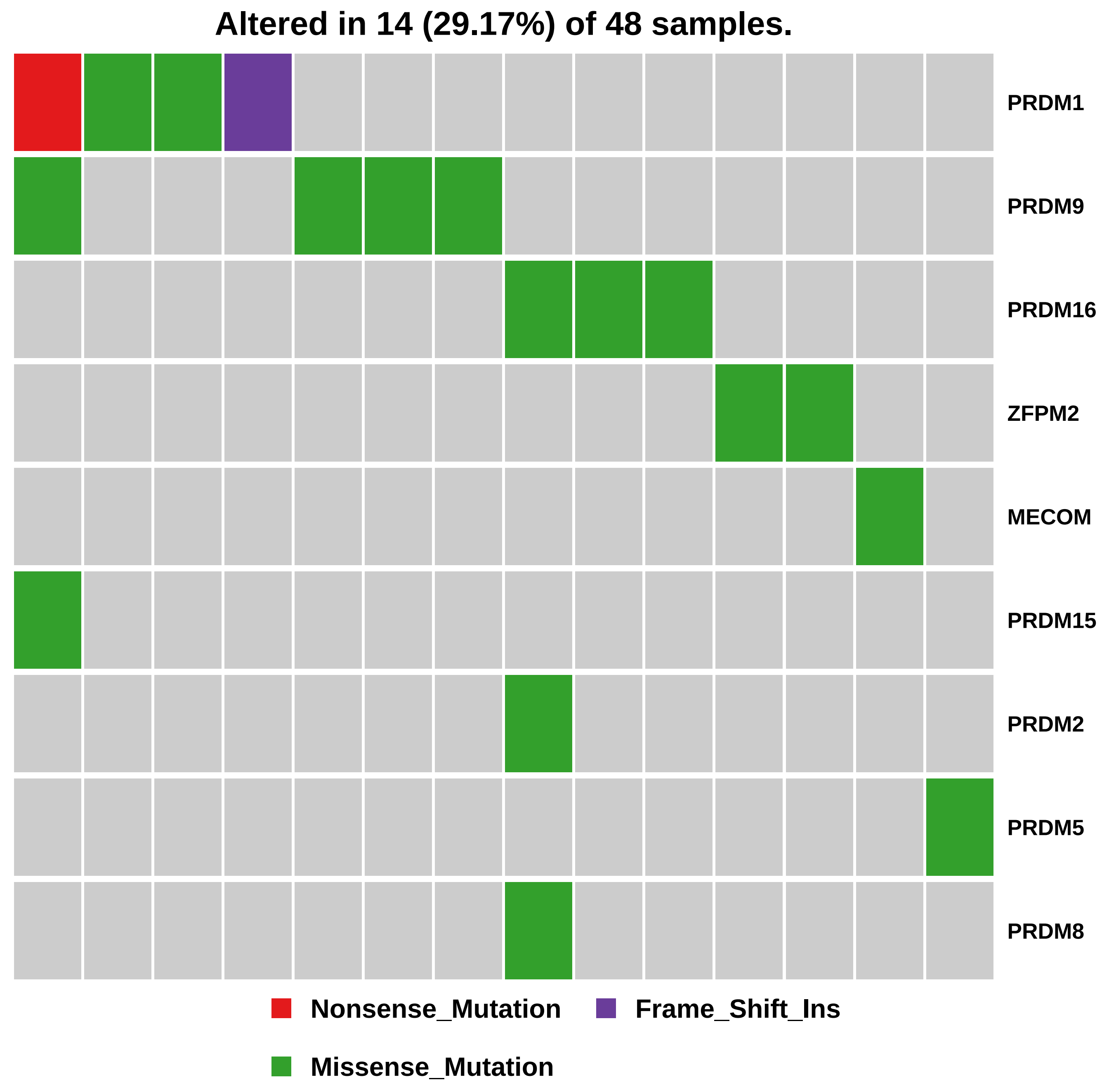
Altered in 14 (29.17%) of 48 samples.
PRDM1
PRDM9
PRDM16
ZFPM2
MECOM
PRDM15
PRDM2
PRDM5
PRDM8
Nonsense_Mutation
Frame_Shift_Ins
Missense_Mutation

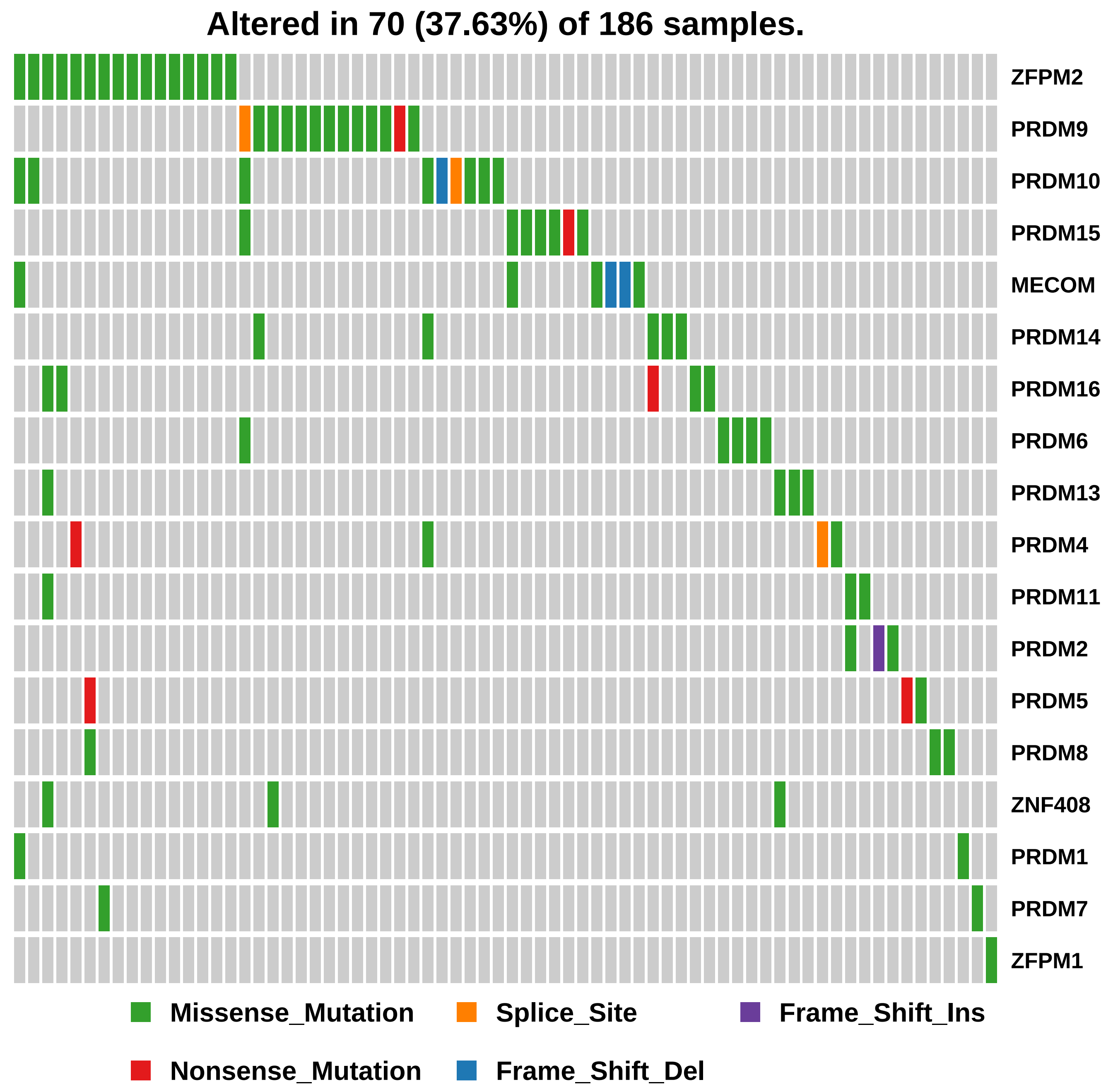
Altered in 70 (37.63%) of 186 samples.
ZFPM2
PRDM9
PRDM10
PRDM15
MECOM
PRDM14
PRDM16
PRDM6
PRDM13
PRDM4
PRDM11
PRDM2
PRDM5
PRDM8
ZNF408
PRDM1
PRDM7
ZFPM1
Missense_Mutation
Splice_Site
Frame_Shift_Ins
Nonsense_Mutation
Frame_Shift_Del

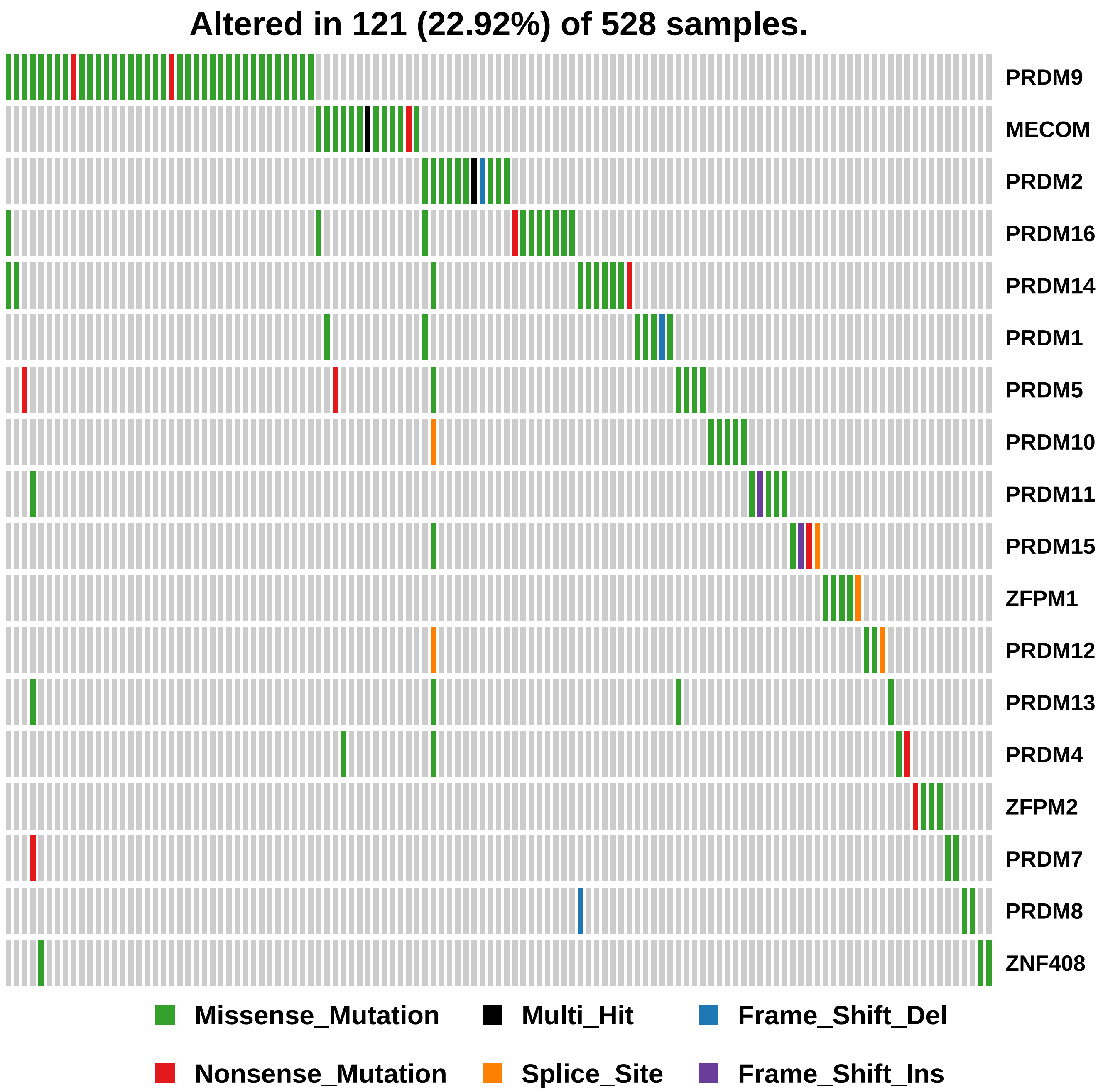
Altered in 121 (22.92%) of 528 samples.
PRDM9
MECOM
PRDM2
PRDM16
PRDM14
PRDM1
PRDM5
PRDM10
PRDM11
PRDM15
ZFPM1
PRDM12
PRDM13
PRDM4
ZFPM2
PRDM7
PRDM8
ZNF408
Missense_Mutation
Multi_Hit
Frame_Shift_Del
Nonsense_Mutation
Splice_Site
Frame_Shift_Ins

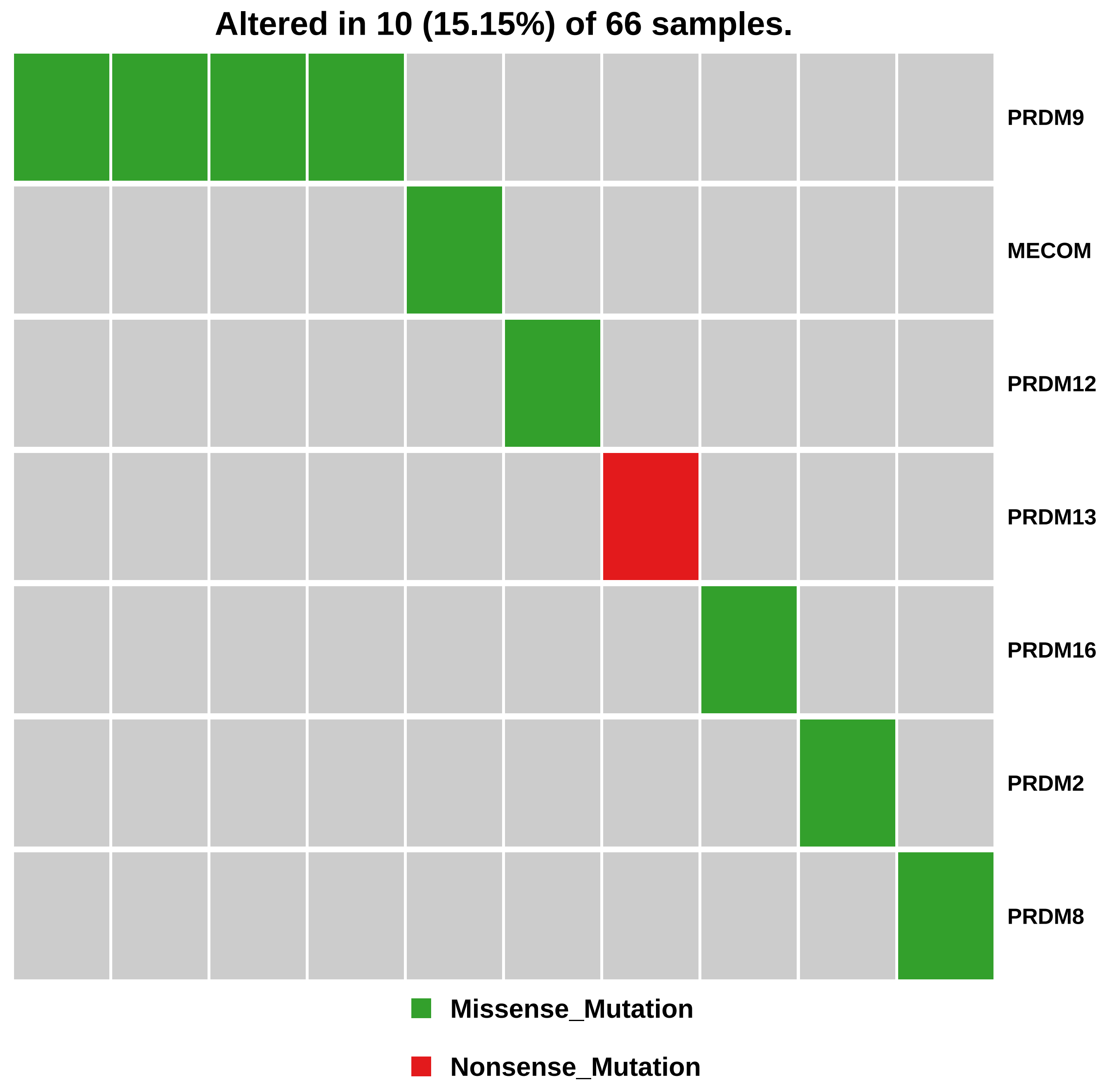
Altered in 10 (15.15%) of 66 samples.
PRDM9
MECOM
PRDM12
PRDM13
PRDM16
PRDM2
PRDM8
Missense_Mutation
Nonsense_Mutation

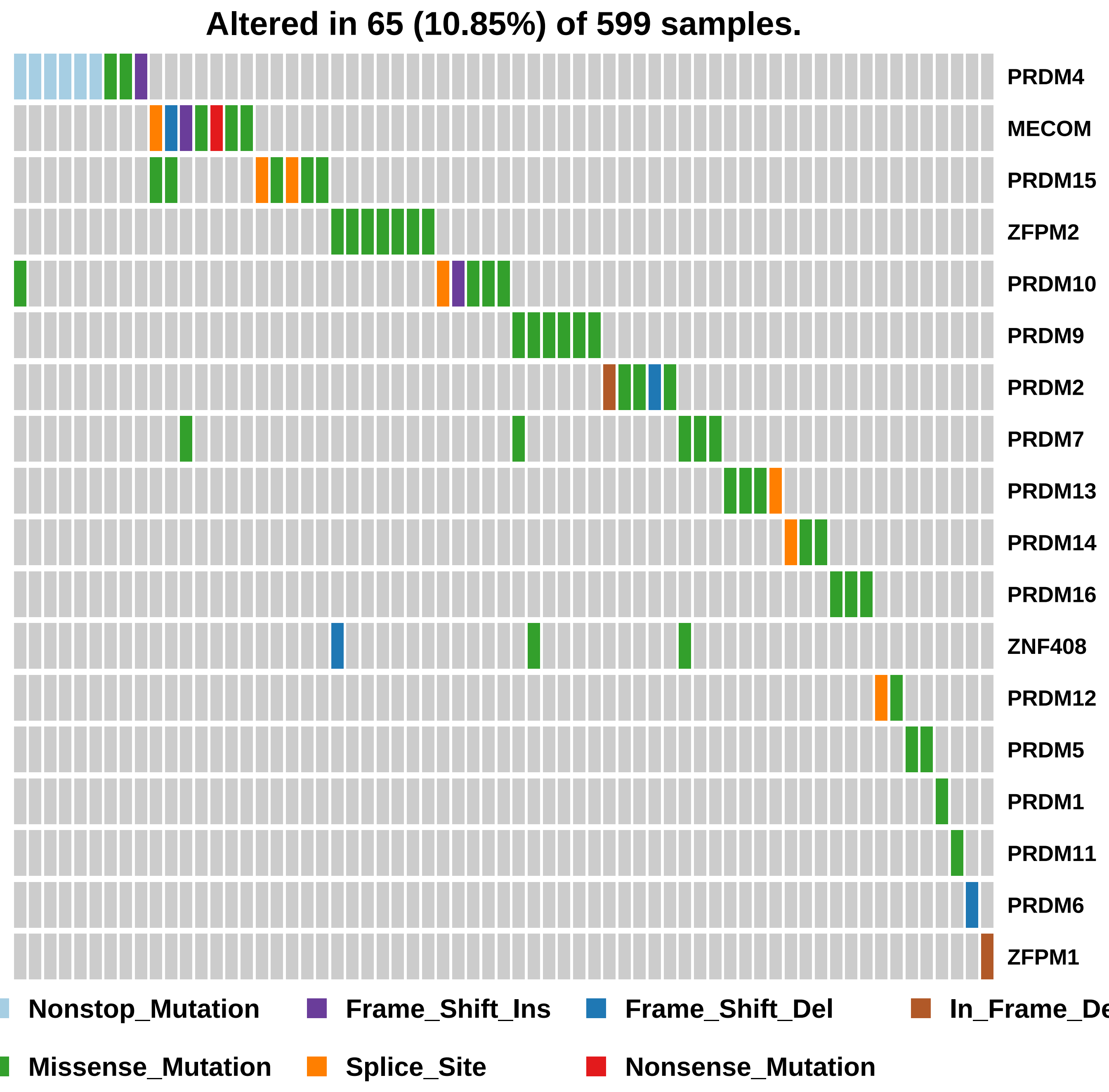
Altered in 65 (10.85%) of 599 samples.
PRDM4
MECOM
PRDM15
ZFPM2
PRDM10
PRDM9
PRDM2
PRDM7
PRDM13
PRDM14
PRDM16
ZNF408
PRDM12
PRDM5
PRDM1
PRDM11
PRDM6
ZFPM1
Nonstop_Mutation
Frame_Shift_Ins
Frame_Shift_Del
Missense_Mutation
Splice_Site
Nonsense_Mutation

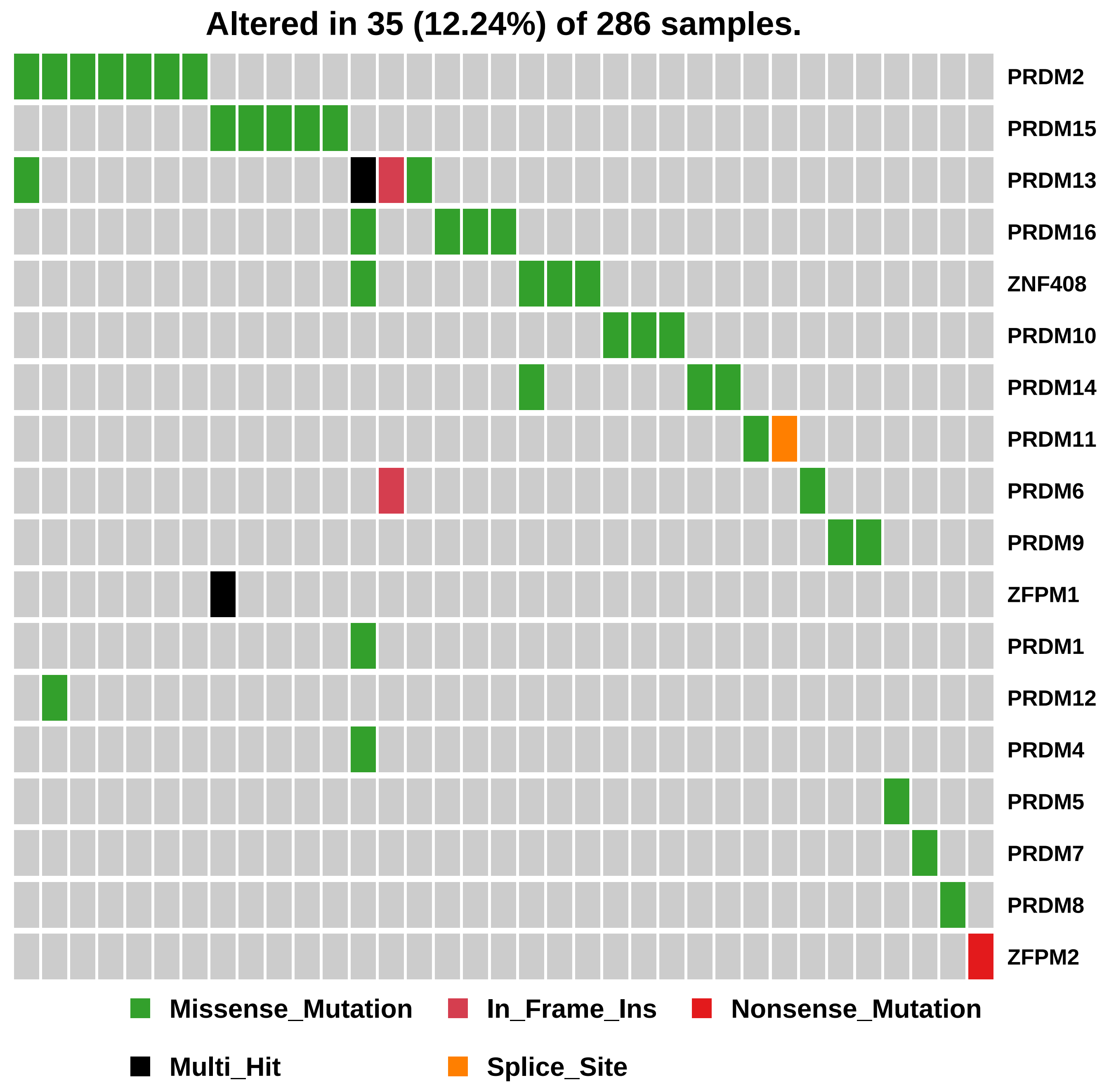

Altered in 35 (12.24%) of 286 samples.
PRDM2
PRDM15
PRDM13
PRDM16
ZNF408
PRDM10
PRDM14
PRDM11
PRDM6
PRDM9
ZFPM1
PRDM1
PRDM12
PRDM4
PRDM5
PRDM7
PRDM8
ZFPM2
Missense_Mutation
In_Frame_Ins
Nonsense_Mutation
Multi_Hit
Splice_Site

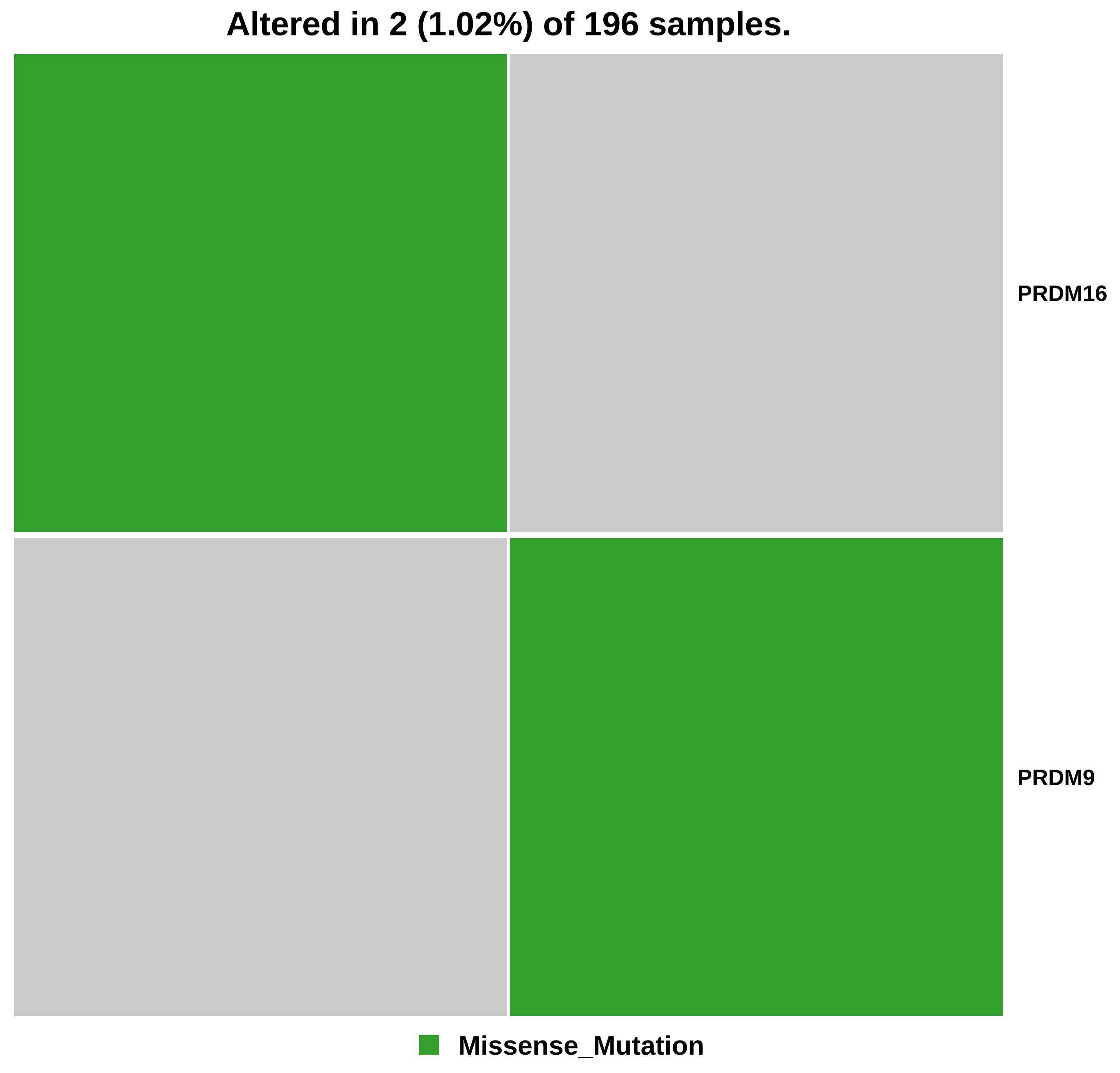
Altered in 2 (1.02%) of 196 samples.
PRDM16
PRDM9
Missense_Mutation

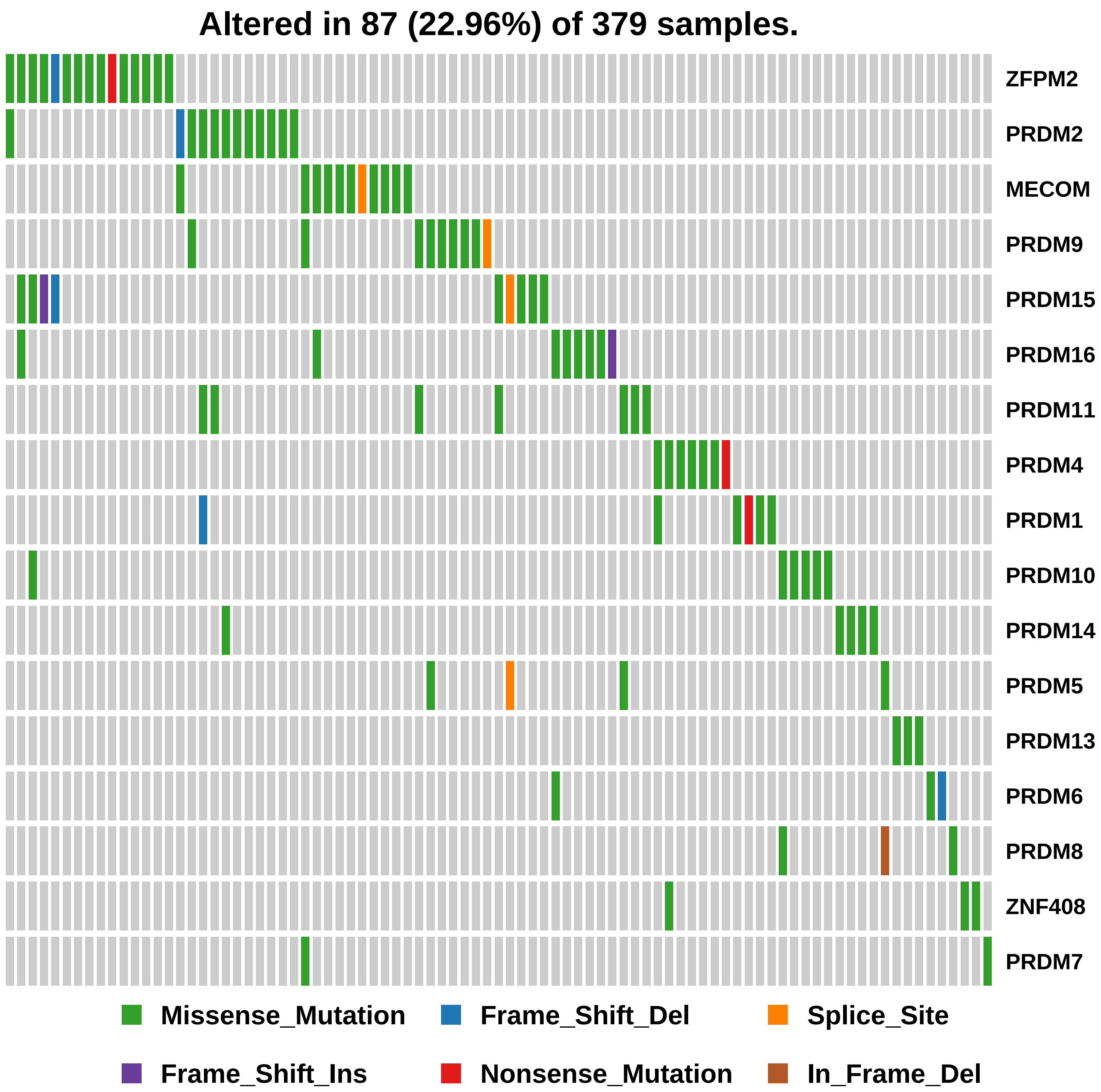
Altered in 87 (22.96%) of 379 samples.
ZFPM2
PRDM2
MECOM
PRDM9
PRDM15
PRDM16
PRDM11
PRDM4
PRDM1
PRDM10
PRDM14
PRDM5
PRDM13
PRDM6
PRDM8
ZNF408
PRDM7
Missense_Mutation
Frame_Shift_Del
Splice_Site
Frame_Shift_Ins
Nonsense_Mutation
In_Frame_Del

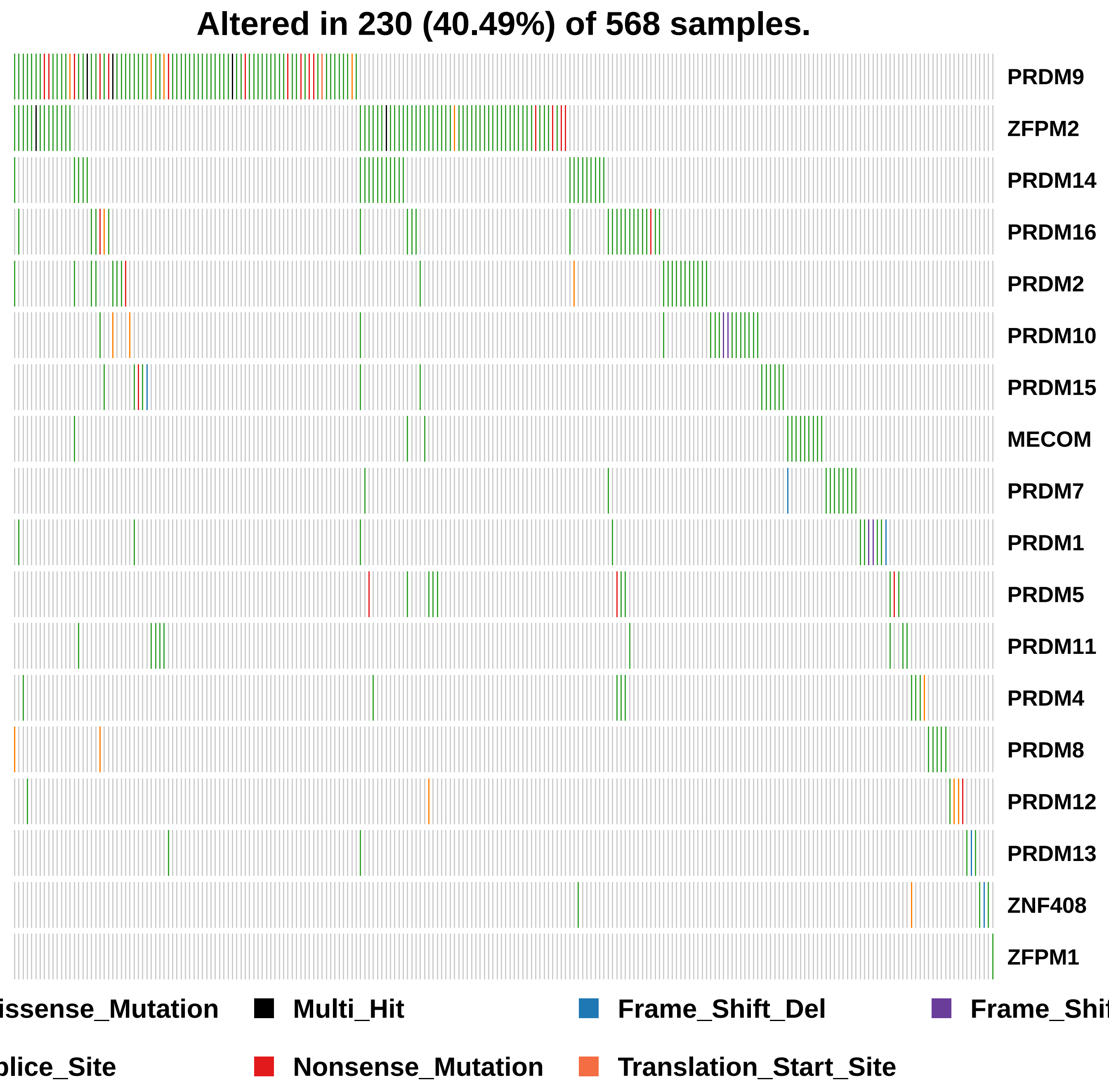
Altered in 230 (40.49%) of 568 samples.
PRDM9
ZFPM2
PRDM14
PRDM16
PRDM2
PRDM10
PRDM15
MECOM
PRDM7
PRDM1
PRDM5
PRDM11
PRDM4
PRDM8
PRDM12
PRDM13
ZNF408
ZFPM1
issense_Mutation
Multi_Hit
Frame_Shift_Del
Frame_Shi
plice_Site
Nonsense_Mutation
Translation_Start_Site

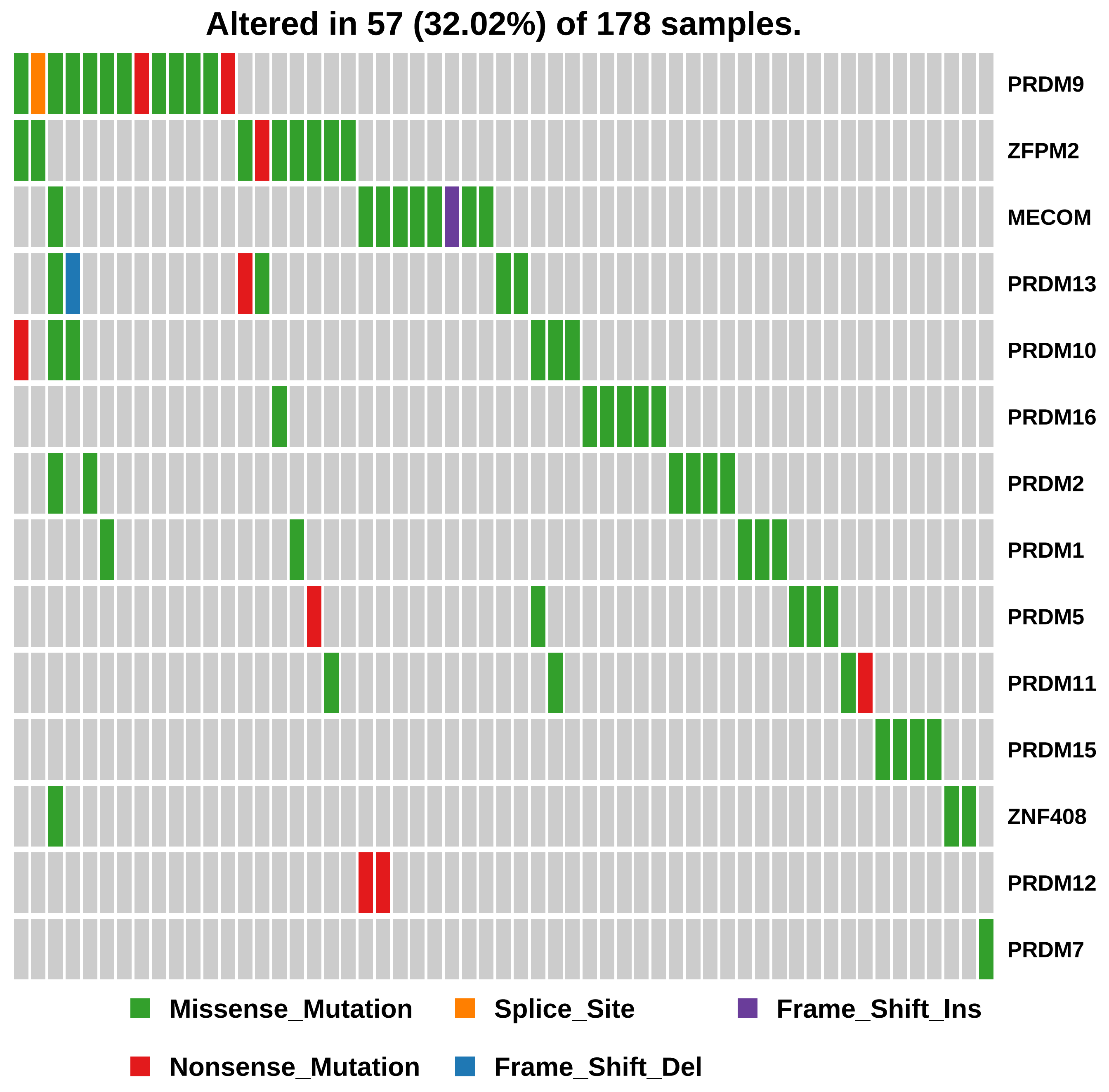

Altered in 57 (32.02%) of 178 samples.
PRDM9
ZFPM2
MECOM
PRDM13
PRDM10
PRDM16
PRDM2
PRDM1
PRDM5
PRDM11
PRDM15
ZNF408
PRDM12
PRDM7
Missense_Mutation
Splice_Site
Frame_Shift_Ins
Nonsense_Mutation
Frame_Shift_Del

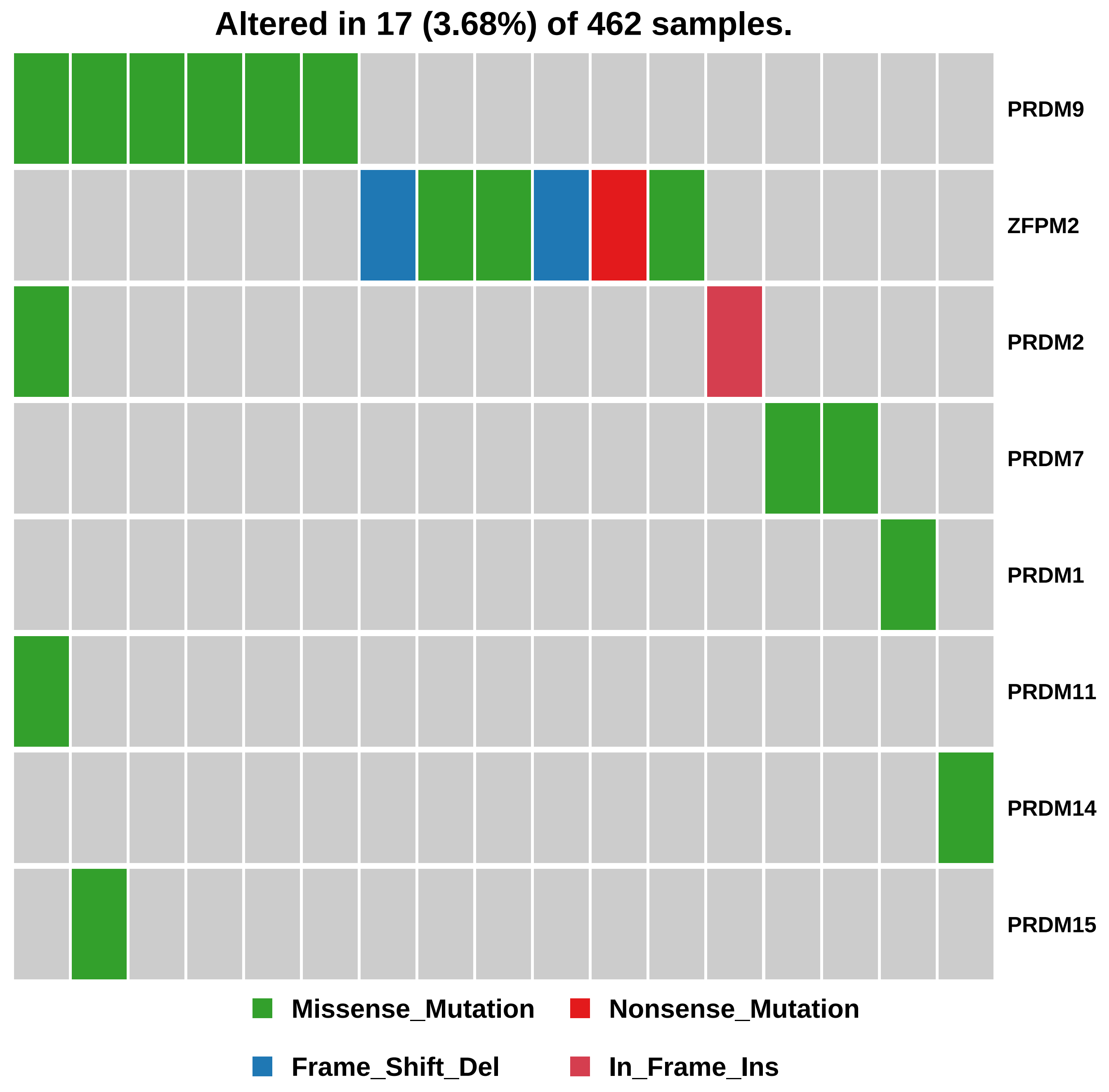
Altered in 17 (3.68%) of 462 samples.
PRDM9
ZFPM2
PRDM2
PRDM7
PRDM1
PRDM11
PRDM14
PRDM15
Missense_Mutation
Nonsense_Mutation
Frame_Shift_Del
In_Frame_Ins

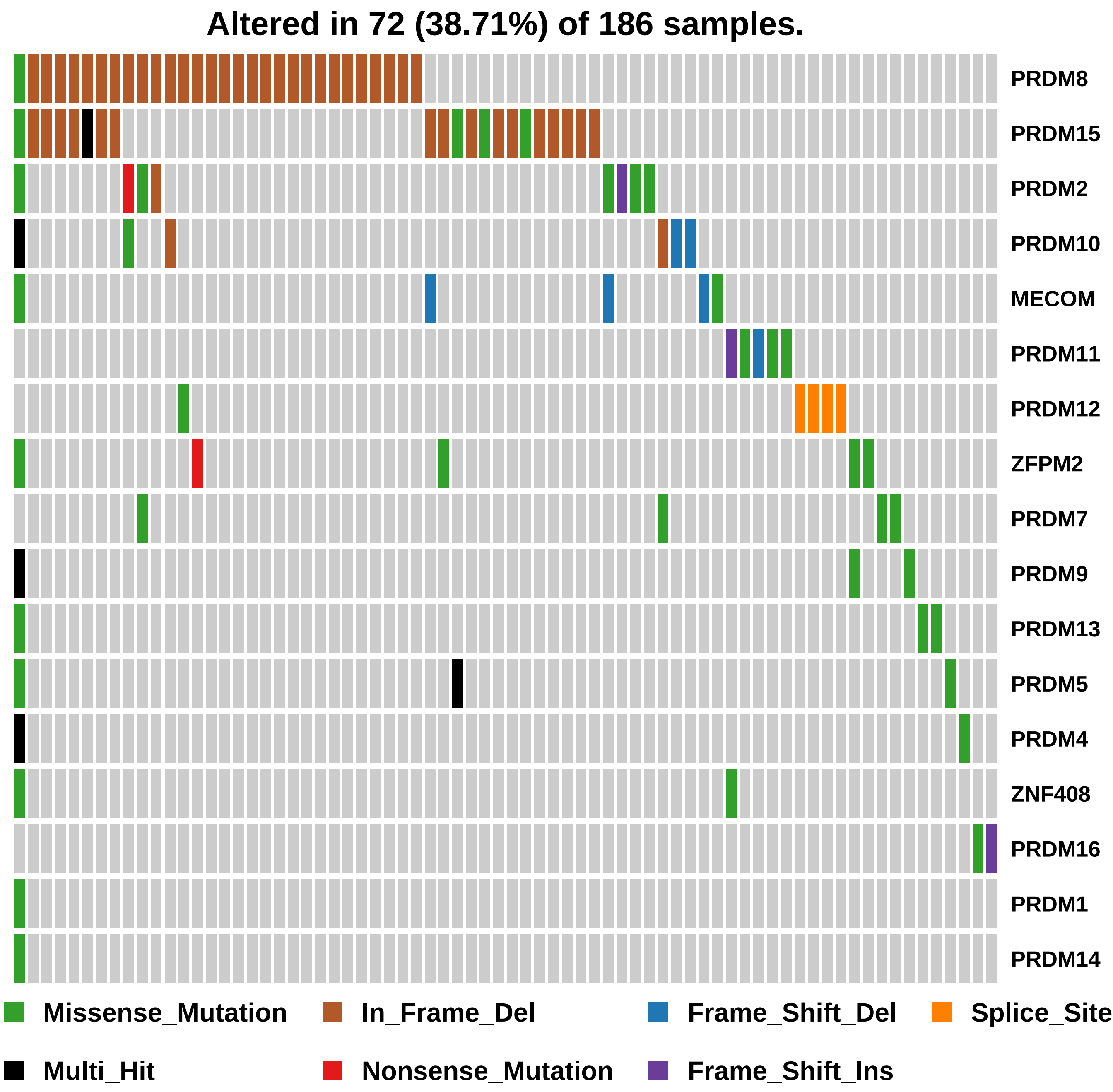
Altered in 72 (38.71%) of 186 samples.
PRDM8
PRDM15
PRDM2
PRDM10
MECOM
PRDM11
PRDM12
ZFPM2
PRDM7
PRDM9
PRDM13
PRDM5
PRDM4
ZNF408
PRDM16
PRDM1
PRDM14
Missense_Mutation
In_Frame_Del
Frame_Shift_Del
Splice_Site
Multi_Hit
Nonsense_Mutation
Frame_Shift_Ins

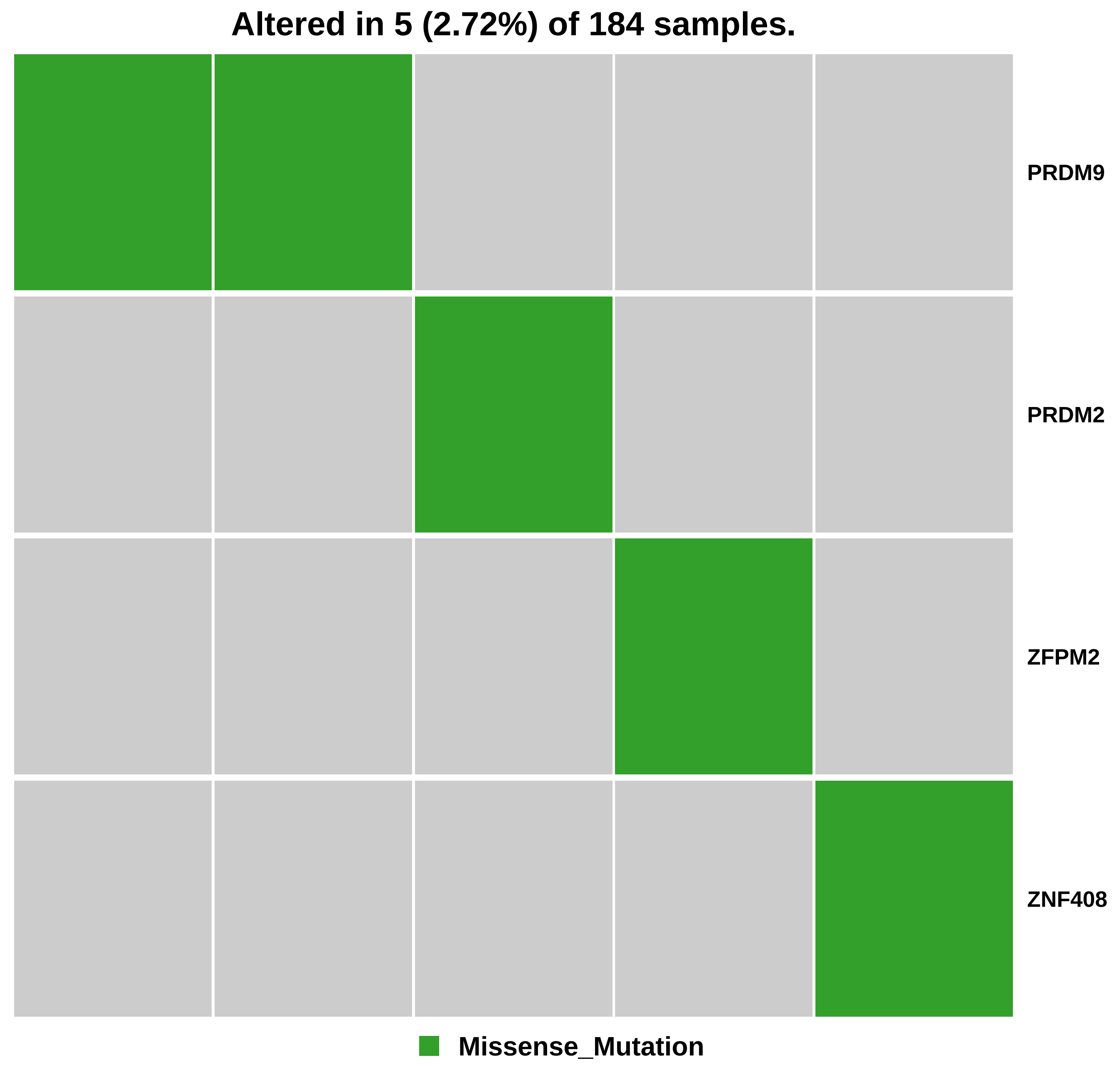
Altered in 5 (2.72%) of 184 samples.
PRDM9
PRDM2
ZFPM2
ZNF408
Missense_Mutation

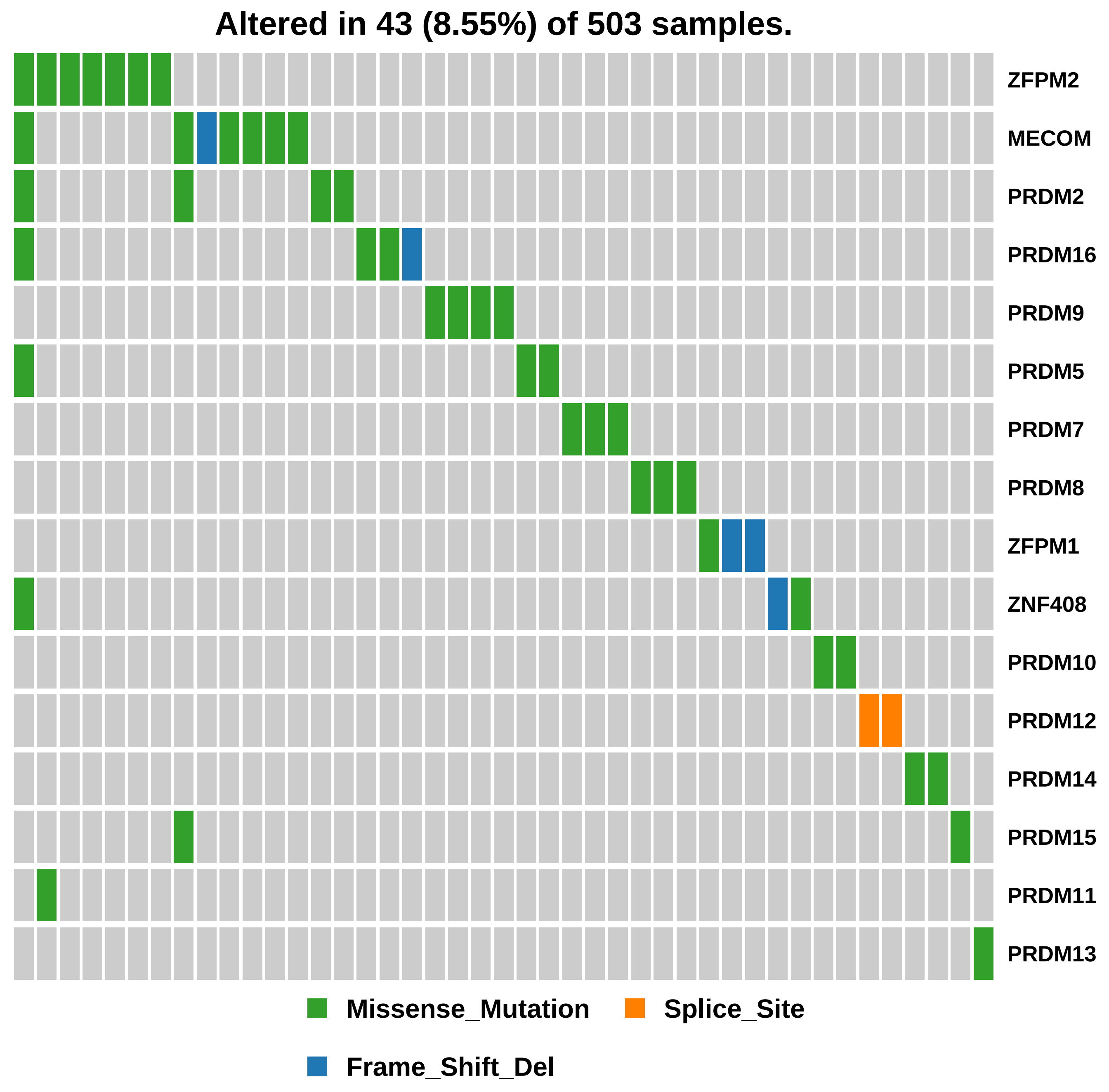

Altered in 43 (8.55%) of 503 samples.
ZFPM2
MECOM
PRDM2
PRDM16
PRDM9
PRDM5
PRDM7
PRDM8
ZFPM1
ZNF408
PRDM10
PRDM12
PRDM14
PRDM15
PRDM11
PRDM13
Missense_Mutation
Splice_Site
Frame_Shift_Del

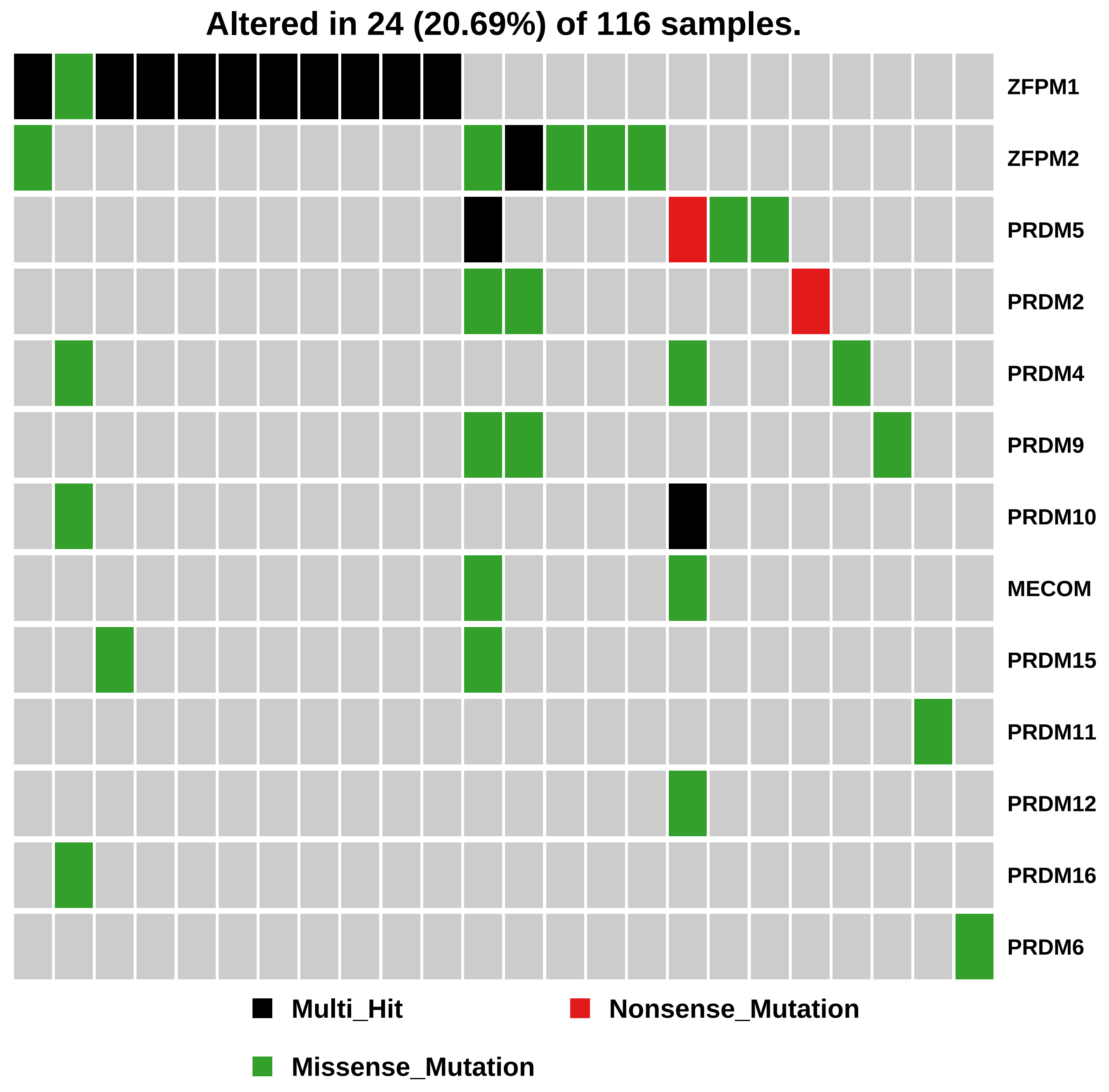
Altered in 24 (20.69%) of 116 samples.
ZFPM1
ZFPM2
PRDM5
PRDM2
PRDM4
PRDM9
PRDM10
MECOM
PRDM15
PRDM11
PRDM12
PRDM16
PRDM6
Multi_Hit
Nonsense_Mutation
Missense_Mutation

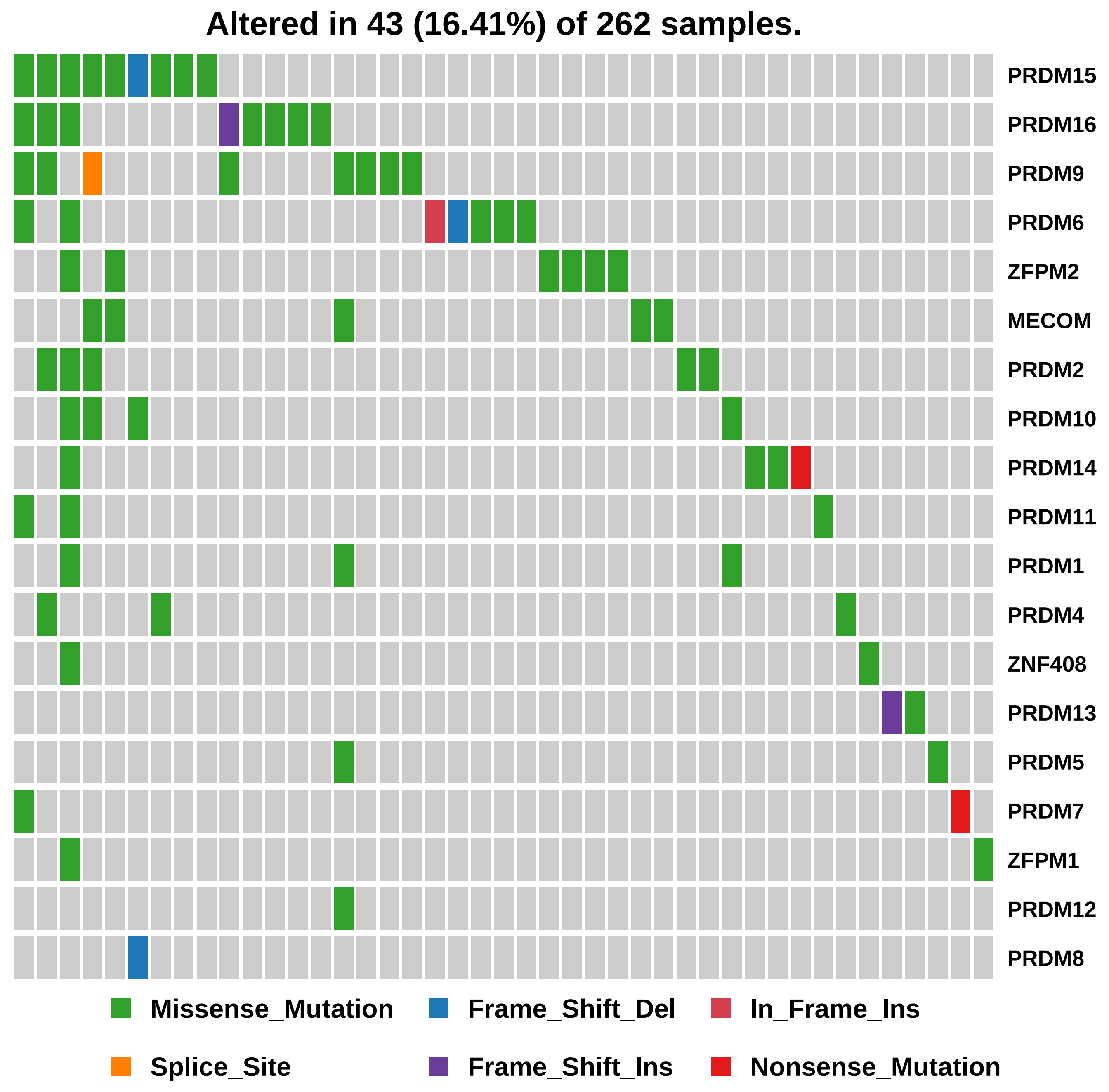
Altered in 43 (16.41%) of 262 samples.
PRDM15
PRDM16
PRDM9
PRDM6
ZFPM2
MECOM
PRDM2
PRDM10
PRDM14
PRDM11
PRDM1
PRDM4
ZNF408
PRDM13
PRDM5
PRDM7
ZFPM1
PRDM12
PRDM8
Missense_Mutation
Frame_Shift_Del
In_Frame_Ins
Splice_Site
Frame_Shift_Ins
Nonsense_Mutation

Altered in 237 (50.21%) of 472 samples.

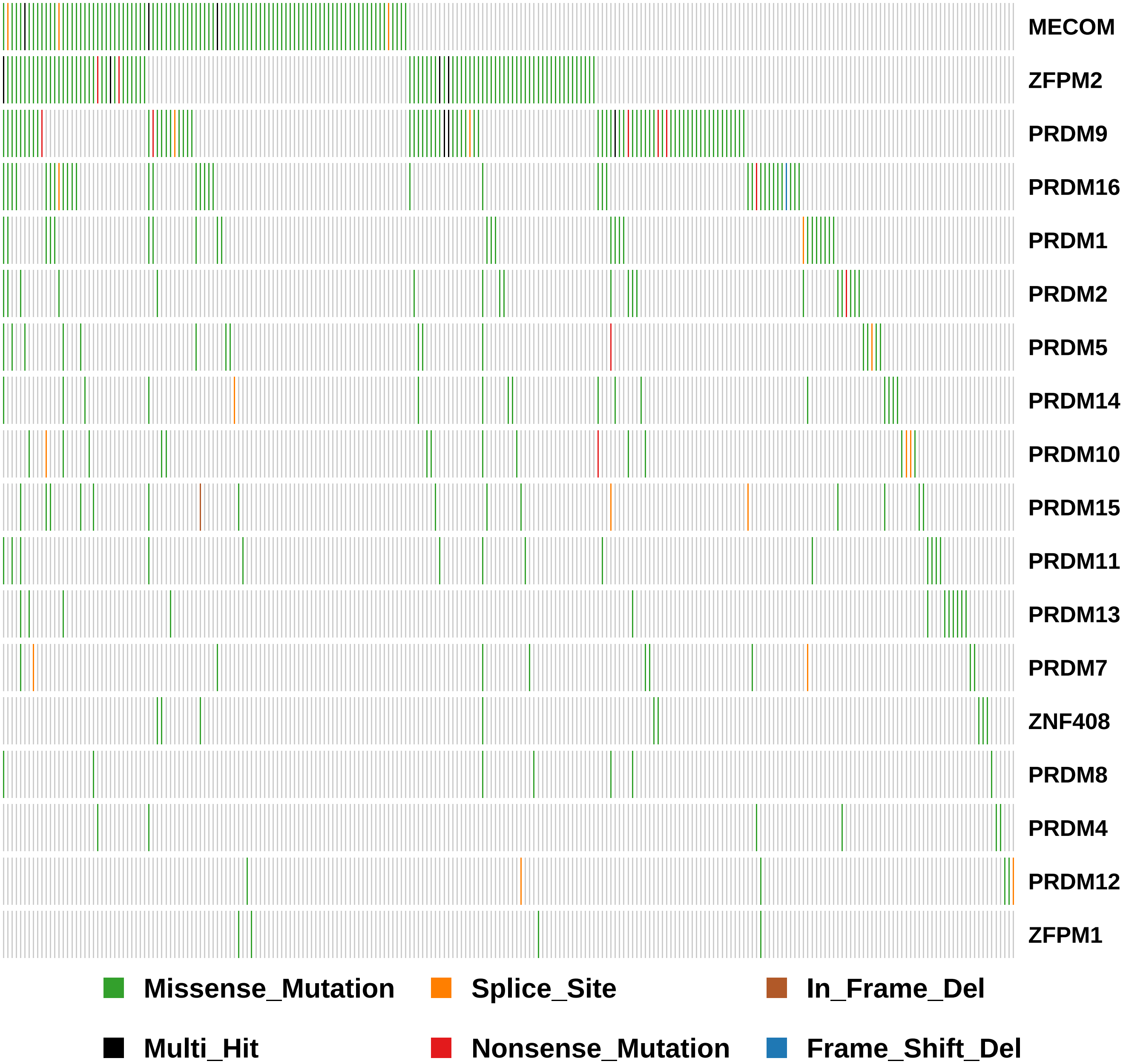

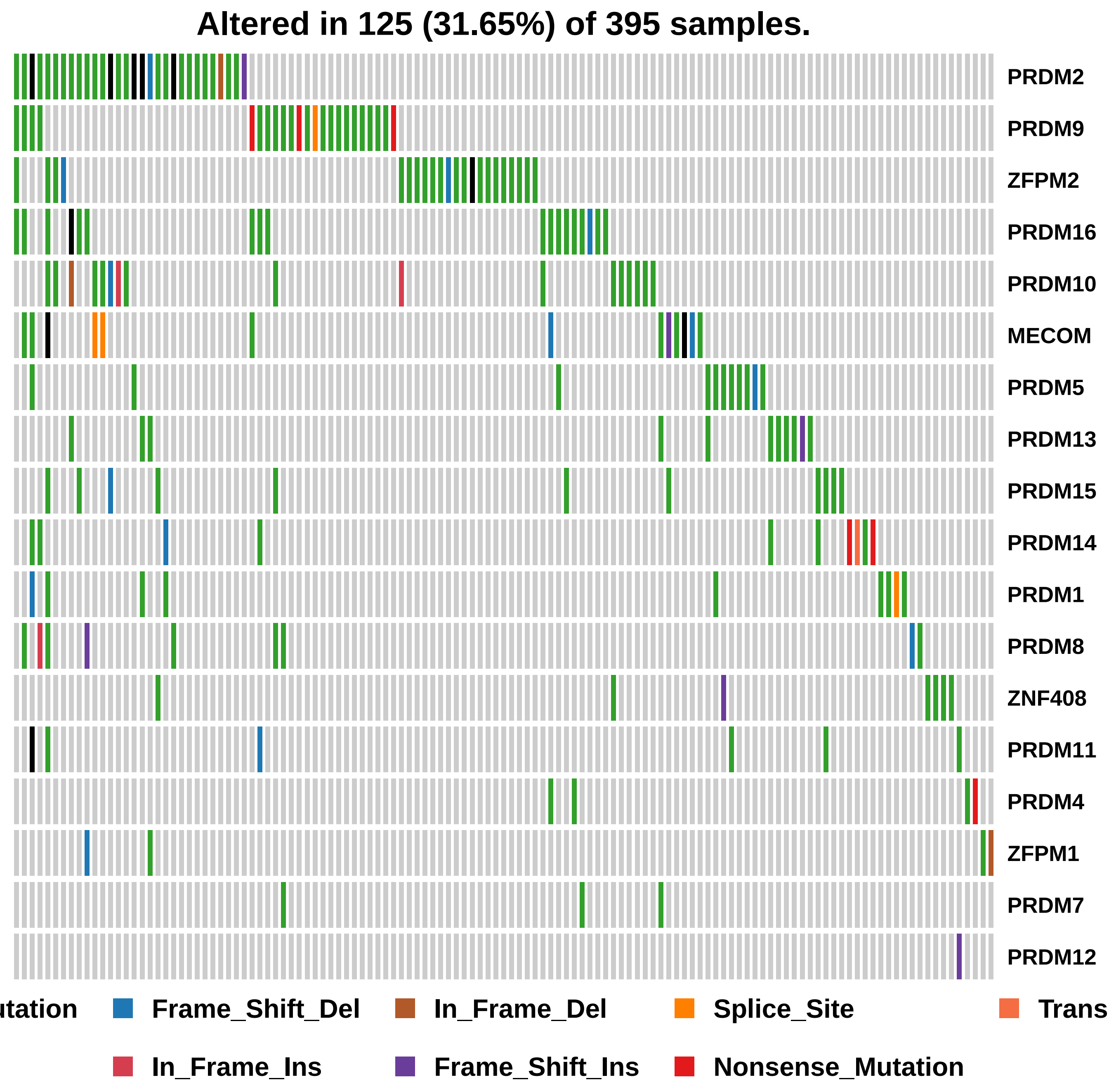
Altered in 125 (31.65%) of 395 samples.
PRDM2
PRDM9
ZFPM2
PRDM16
PRDM10
MECOM
PRDM5
PRDM13
PRDM15
PRDM14
PRDM1
PRDM8
ZNF408
PRDM11
PRDM4
ZFPM1
PRDM7
PRDM12
utation
Frame_Shift_Del
In_Frame_Del
Splice_Site
Trans
In_Frame_Ins
Frame_Shift_Ins
Nonsense_Mutation

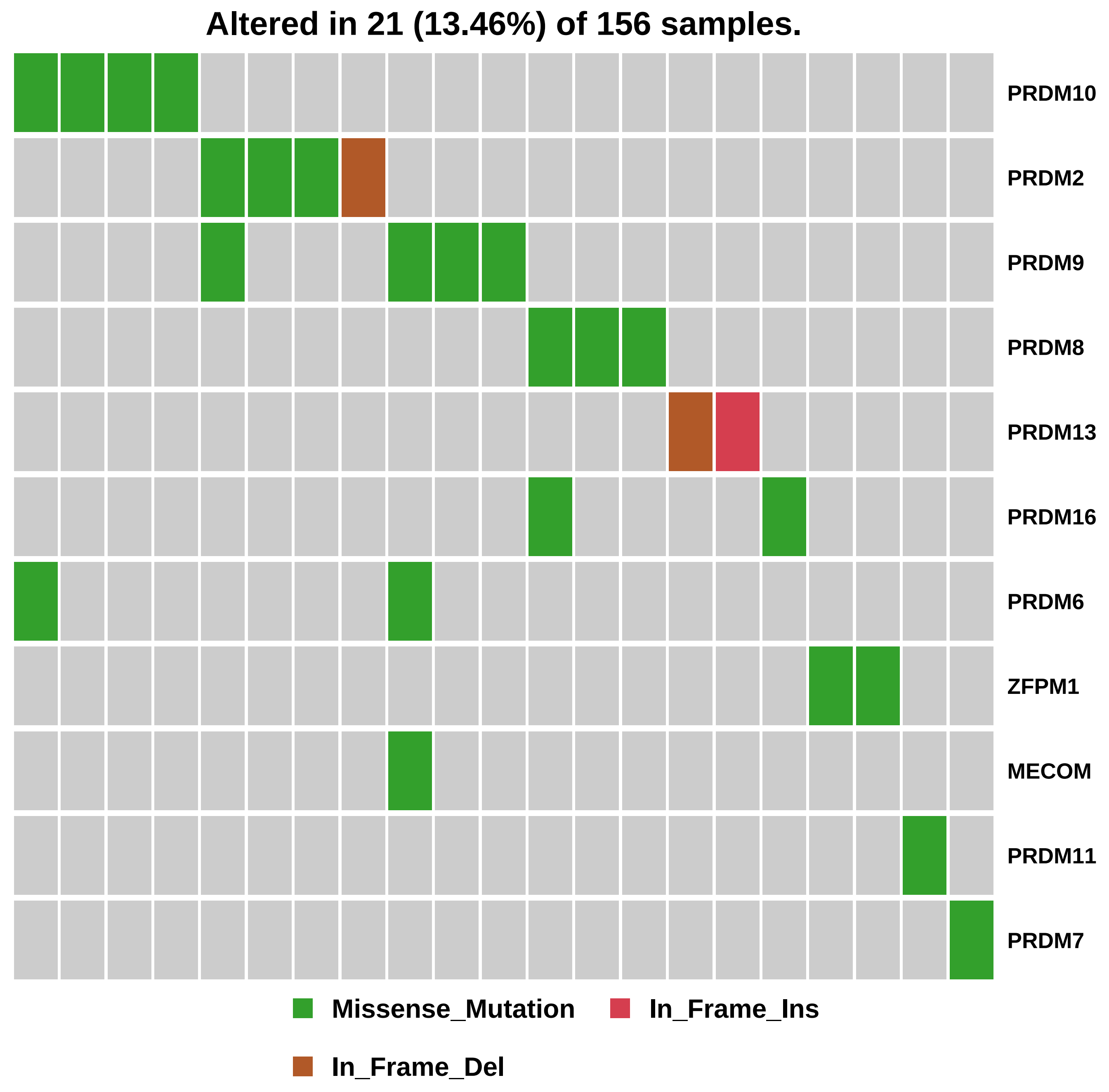
Altered in 21 (13.46%) of 156 samples.
PRDM10
PRDM2
PRDM9
PRDM8
PRDM13
PRDM16
PRDM6
ZFPM1
MECOM
PRDM11
PRDM7
Missense_Mutation
In_Frame_Ins
In_Frame_Del

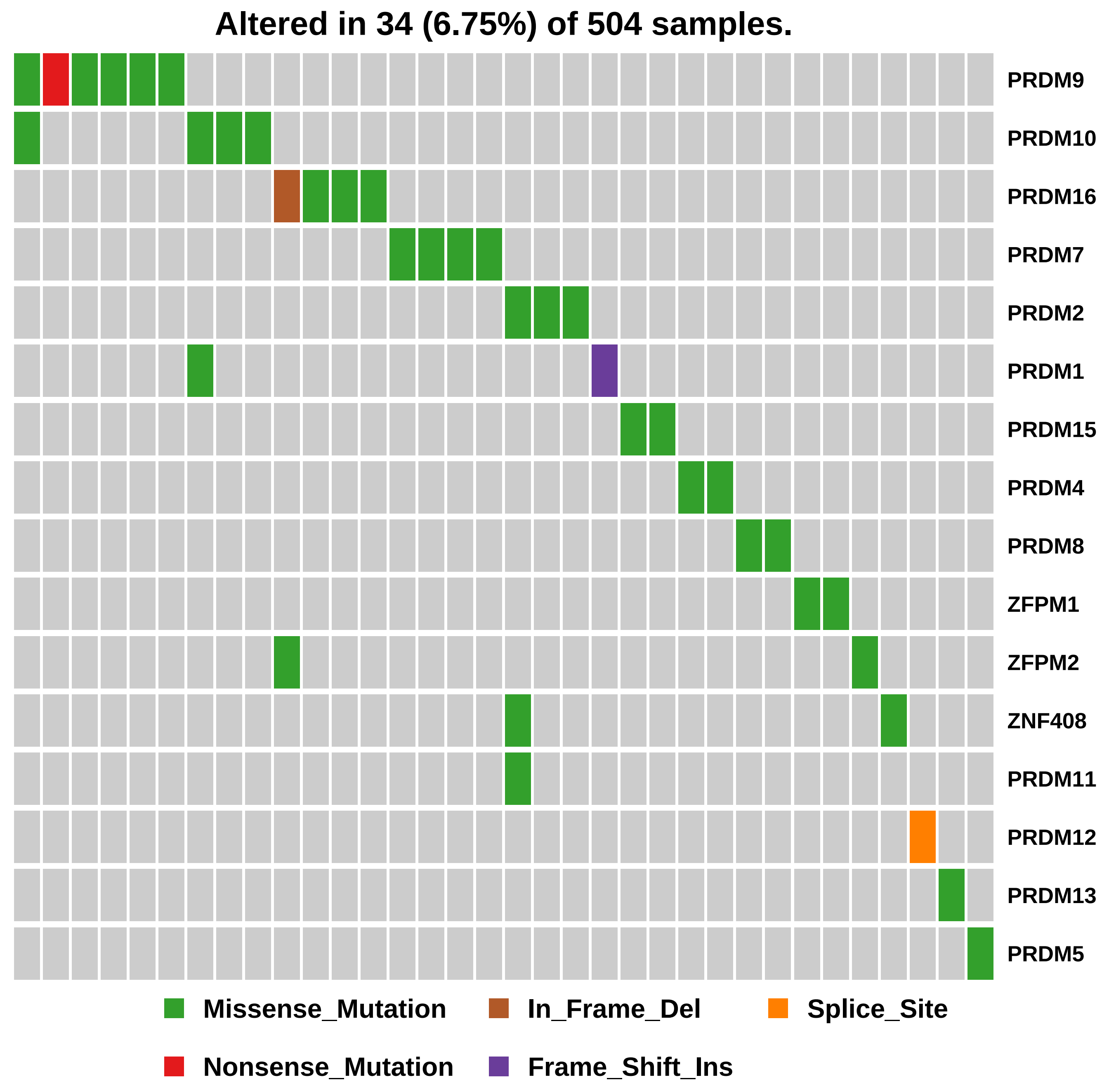
Altered in 34 (6.75%) of 504 samples.
PRDM9
PRDM10
PRDM16
PRDM7
PRDM2
PRDM1
PRDM15
PRDM4
PRDM8
ZFPM1
ZFPM2
ZNF408
PRDM11
PRDM12
PRDM13
PRDM5
Missense_Mutation
In_Frame_Del
Splice_Site
Nonsense_Mutation
Frame_Shift_Ins

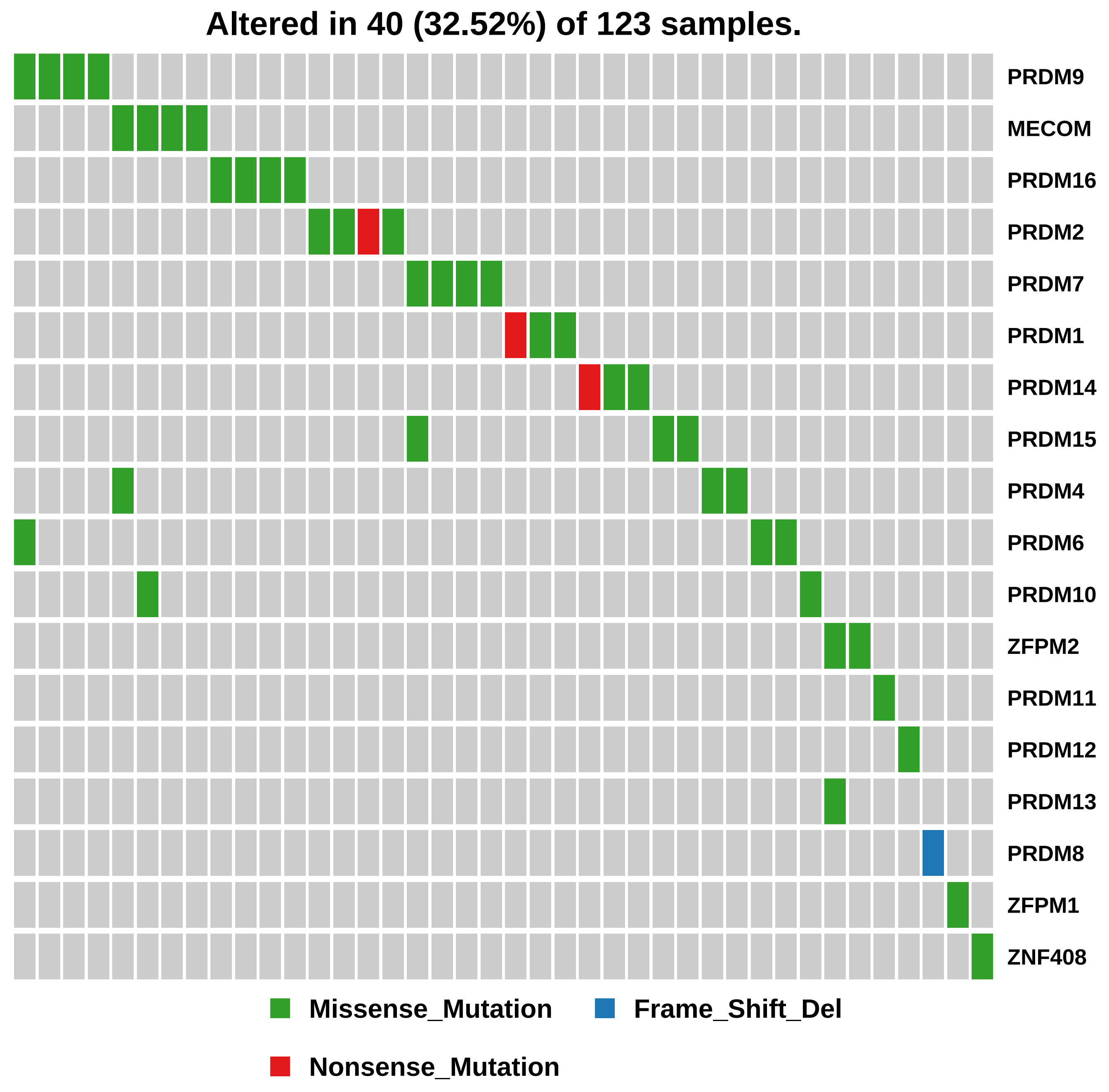

Altered in 40 (32.52%) of 123 samples.
PRDM9
MECOM
PRDM16
PRDM2
PRDM7
PRDM1
PRDM14
PRDM15
PRDM4
PRDM6
PRDM10
ZFPM2
PRDM11
PRDM12
PRDM13
PRDM8
ZFPM1
ZNF408
Missense_Mutation
Frame_Shift_Del
Nonsense_Mutation

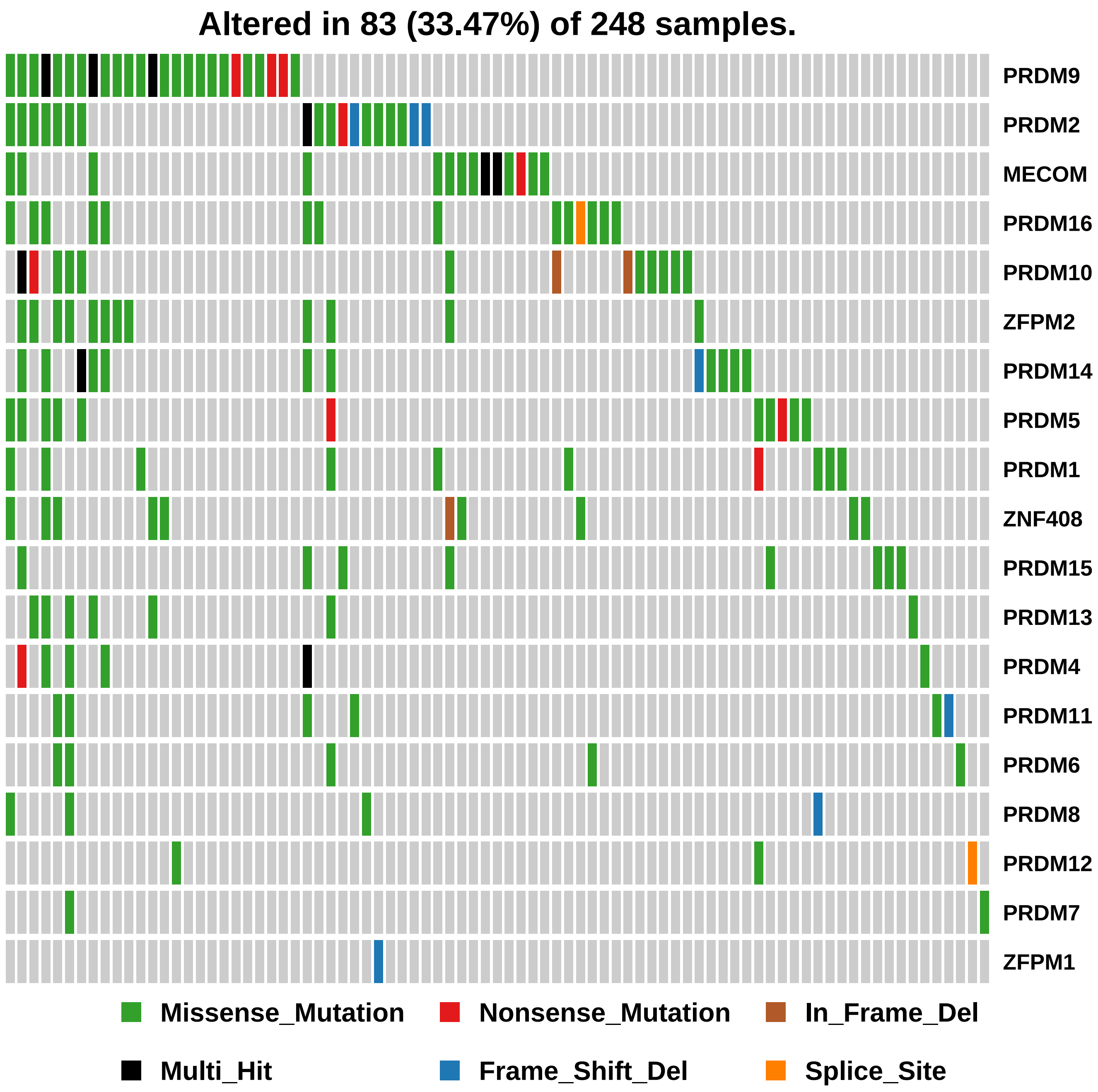
Altered in 83 (33.47%) of 248 samples.
PRDM9
PRDM2
MECOM
PRDM16
PRDM10
ZFPM2
PRDM14
PRDM5
PRDM1
ZNF408
PRDM15
PRDM13
PRDM4
PRDM11
PRDM6
PRDM8
PRDM12
PRDM7
ZFPM1
Missense_Mutation
Nonsense_Mutation
In_Frame_Del
Multi_Hit
Frame_Shift_Del
Splice_Site

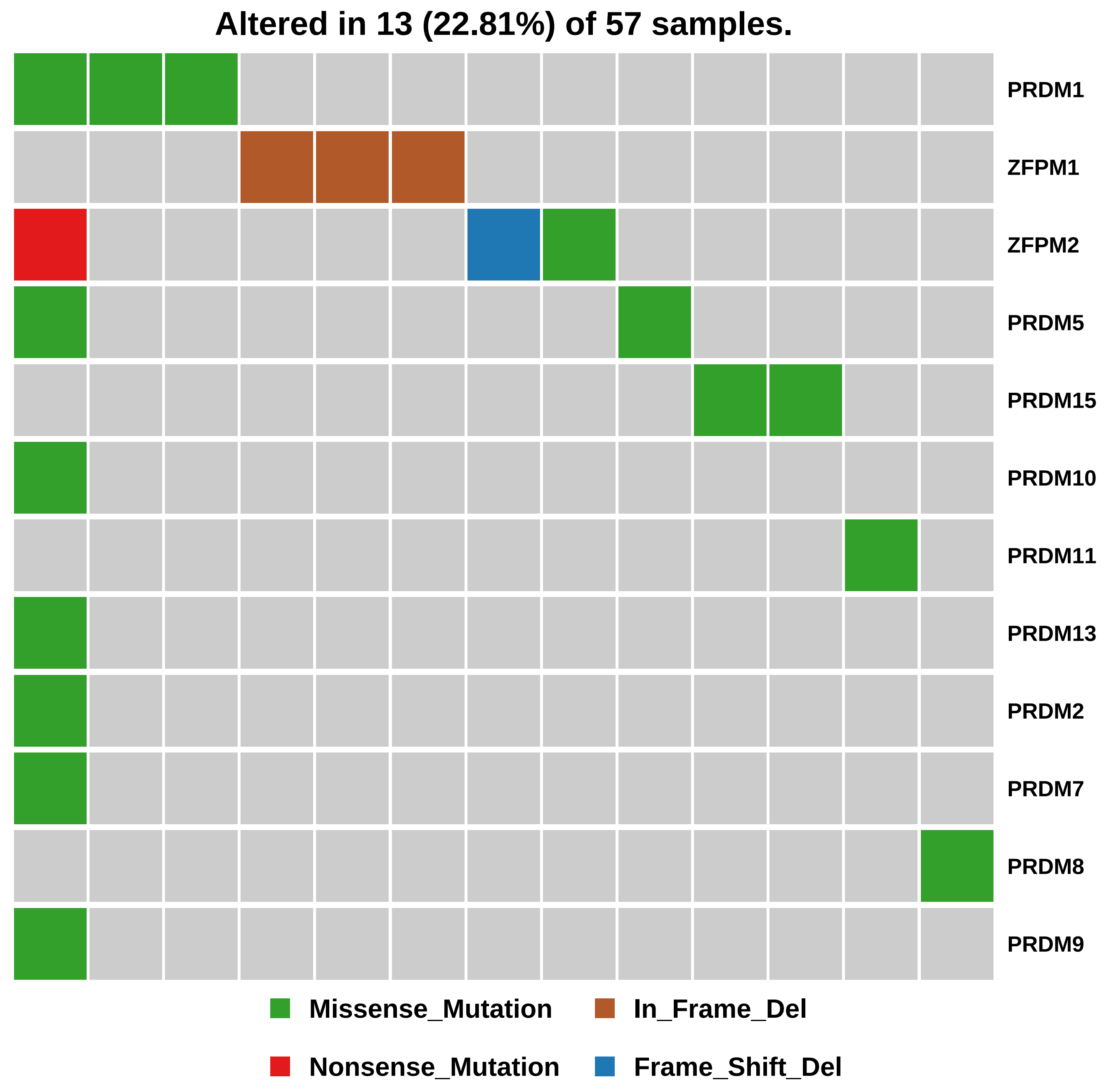
Altered in 13 (22.81%) of 57 samples.
PRDM1
ZFPM1
ZFPM2
PRDM5
PRDM15
PRDM10
PRDM11
PRDM13
PRDM2
PRDM7
PRDM8
PRDM9
Missense_Mutation
In_Frame_Del
Nonsense_Mutation
Frame_Shift_Del

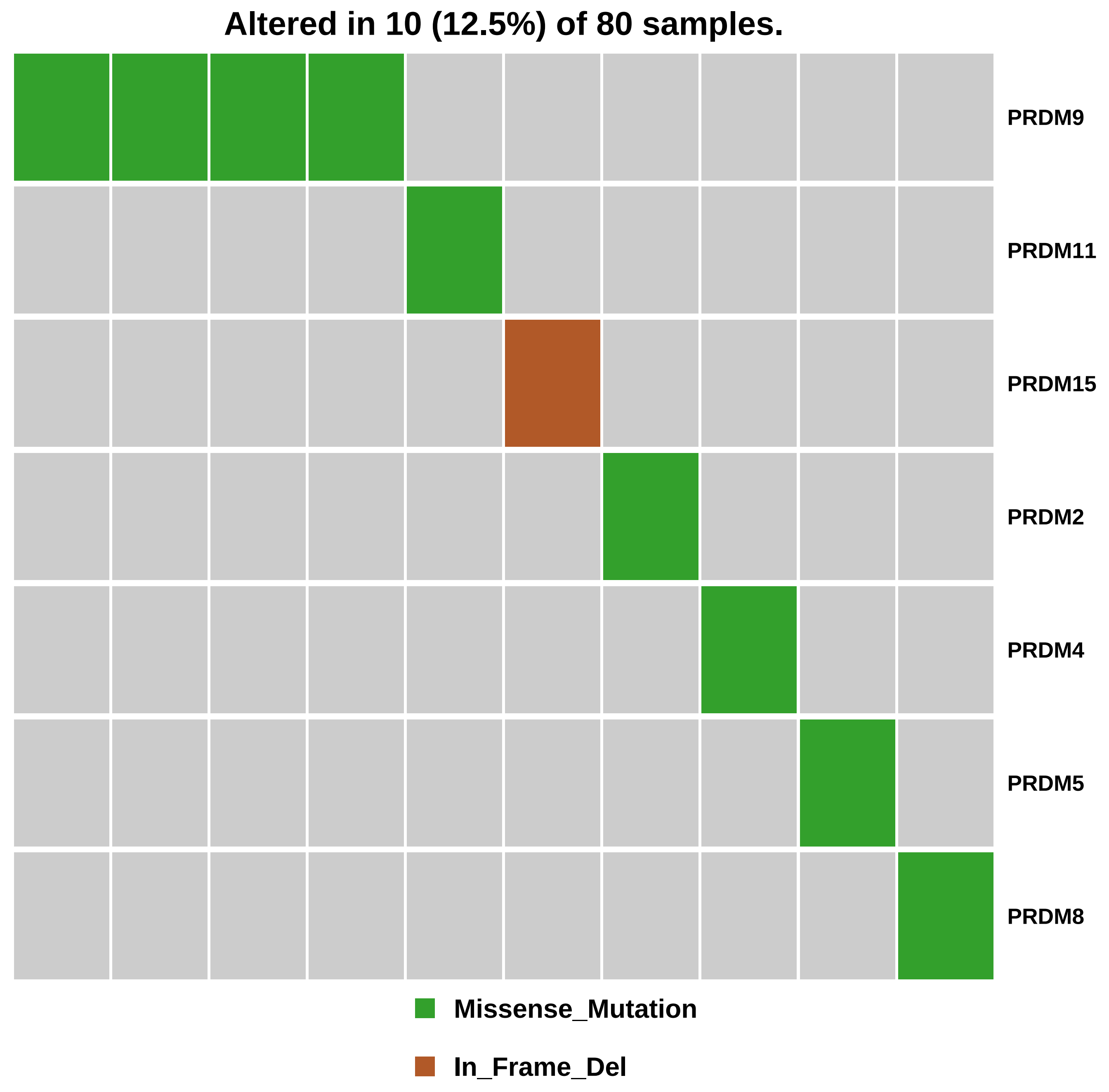
Altered in 10 (12.5%) of 80 samples.
PRDM9
PRDM11
PRDM15
PRDM2
PRDM4
PRDM5
PRDM8
Missense_Mutation
In_Frame_Del
