# Supplementary material for: PR/SET Domain Family and Cancer: Novel Insights from The Cancer Genome Atlas
Source: Int J Mol Sci. 2018 Oct 19;19(10):3250. doi: 10.3390/ijms19103250 (PMC6214140; doi:10.3390/ijms19103250)
Supplement: Supplementary file 1 [file ijms-19-03250-s001.zip › SUPPLEMENTARY/Table_S1.docx]

**Tab. S1** List of cancer types and number of patients (*n*) analyzed from TCGA (The Cancer Genome Atlas) [25]

| **Abbreviation** | **Cancer Type** | **Mutation Analysis n** | **Expression Analysis n (Paired)** |
| --- | --- | --- | --- |
| ACC | Adrenocortical carcinoma | 92 | - |
| BLCA | Bladder cancer | 412 | 19 |
| BRCA | Breast cancer | 1098 | 57 |
| CESC | Cervical squamous cell carcinoma and endocervical adenocarcinoma | 308 | 3 |
| CHOL | Cholangiocarcinoma | 51 | 9 |
| COAD | Colon adenocarcinoma | 463 | 26 |
| DLBC | Lymphoid neoplasm diffuse large B-cell lymphoma | 58 | - |
| ESCA | Esophageal carcinoma | 185 | 13 |
| GBM | Glioblastoma | 617 | 5 |
| HNSC | Head and neck squamous cell carcinoma | 528 | 43 |
| KICH | Kidney chromophobe carcinoma | 113 | 25 |
| KIRC | Kidney renal clear cell carcinoma | 537 | 72 |
| KIRP | Kidney renal papillary cell carcinoma | 291 | 32 |
| LAML | Acute myeloid leukemia | 200 | - |
| LIHC | Liver hepatocarcinoma | 377 | 50 |
| LUAD | Lung adenocarcinoma | 585 | 58 |
| LUSC | Lung squamous cell carcinoma | 504 | 51 |
| OV | Ovarian cancer | 608 | - |
| PAAD | Pancreas adenocarcinoma | 185 | 51 |
| PCPG | Pheochromocytoma and paraganglioma | 179 | 4 |
| PRAD | Prostate adenocarcinoma | 500 | 3 |
| READ | Rectum adenocarcinoma | 172 | 52 |
| SARC | Sarcoma | 261 | 2 |
| SKCM | Skin cutaneous melanoma | 470 | - |
| STAD | Stomach adenocarcinoma | 478 | - |
| TGCT | Testicular germ cell tumors | 150 | - |
| THCA | Thyroid cancer | 507 | 57 |
| THYM | Thymoma | 124 | 2 |
| UCEC | Uterine corpus endometrial carcinoma | 560 | 7 |
| UCS | Uterine carcinosarcoma | 57 | - |
| UVM | Uveal melanoma | 80 | - |
