# Supplementary material for: PR/SET Domain Family and Cancer: Novel Insights from The Cancer Genome Atlas
Source: Int J Mol Sci. 2018 Oct 19;19(10):3250. doi: 10.3390/ijms19103250 (PMC6214140; doi:10.3390/ijms19103250)
Supplement: Supplementary file 1 [file ijms-19-03250-s001.zip › SUPPLEMENTARY/Table_S2.docx]

| **Genes** | **Deleterious Missense mutations** | **Tolerated Missense mutations** |
| --- | --- | --- |
| *PRDM1* | 107 | 56 |
| *PRDM2* | 193 | 106 |
| *MECOM* | 240 | 100 |
| *PRDM4* | 73 | 31 |
| *PRDM5* | 109 | 44 |
| *PRDM6* | 29 | 7 |
| *PRDM7* | 24 | 40 |
| *PRDM8* | 43 | 19 |
| *PRDM9* | 330 | 194 |
| *PRDM10* | 161 | 32 |
| *PRDM11* | 91 | 23 |
| *PRDM12* | 25 | 3 |
| *PRDM13* | 80 | 33 |
| *PRDM14* | 111 | 51 |
| *PRDM15* | 140 | 41 |
| *PRDM16* | 227 | 78 |
| *ZNF408* | 46 | 33 |
| *ZFPM1* | 28 | 19 |
| *ZFPM2* | 189 | 138 |
