# Supplementary figures and images for: PR/SET Domain Family and Cancer: Novel Insights from The Cancer Genome Atlas
Source: Int J Mol Sci. 2018 Oct 19;19(10):3250. doi: 10.3390/ijms19103250 (PMC6214140; doi:10.3390/ijms19103250)

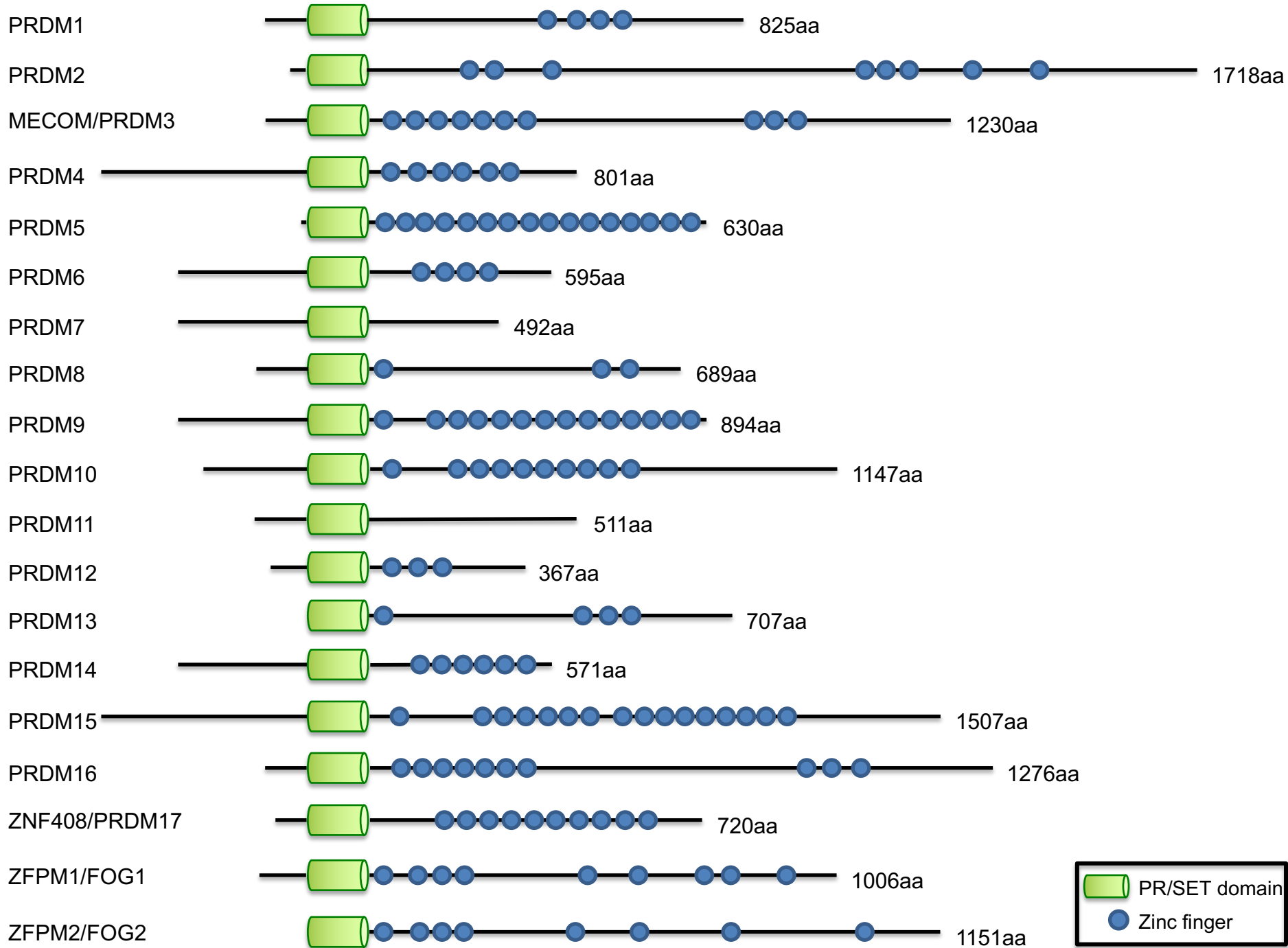

Supplement: Supplementary file 1 [file ijms-19-03250-s001.zip › SUPPLEMENTARY/Figure_S1.pdf]

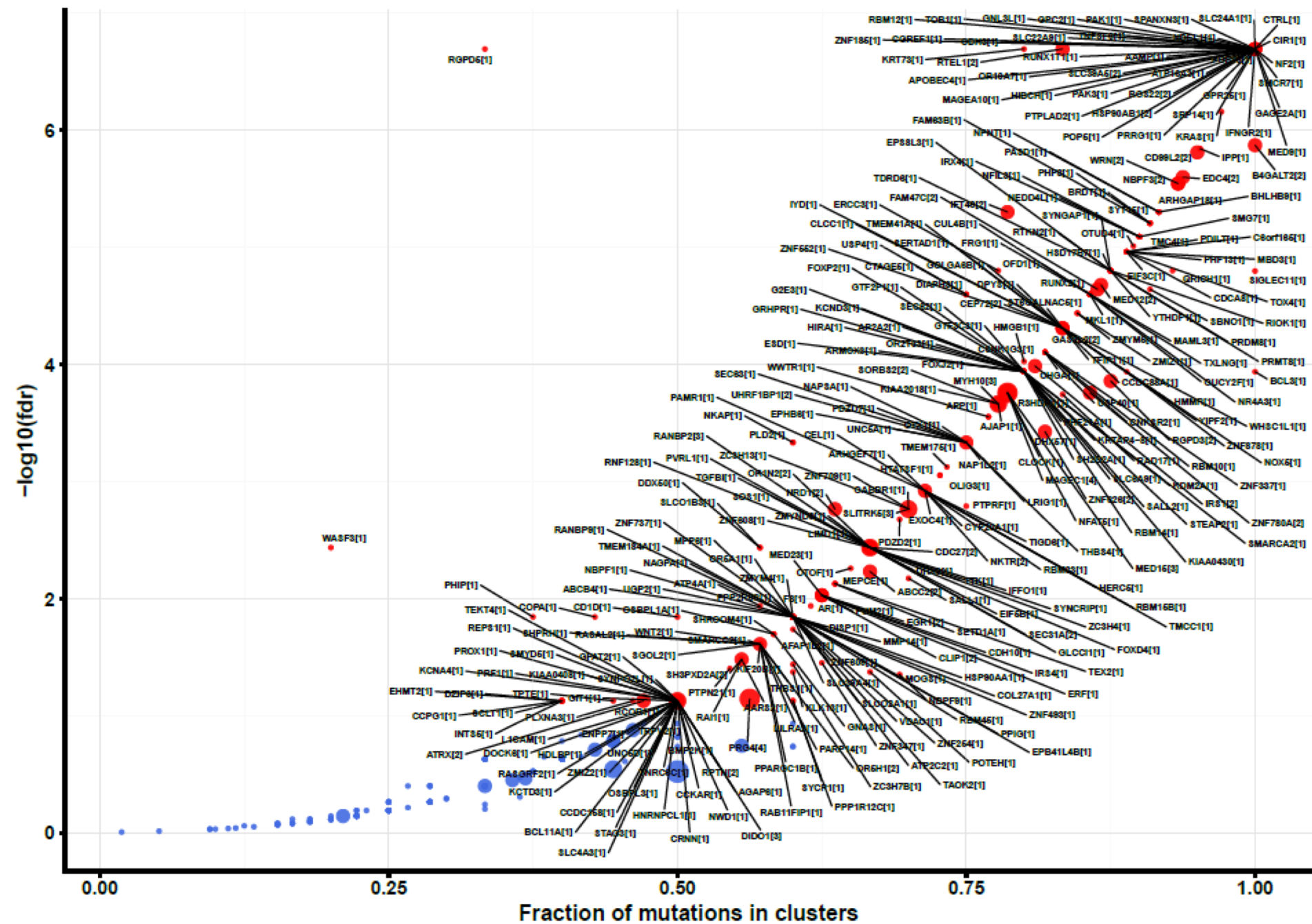

Supplement: Supplementary file 1 [file ijms-19-03250-s001.zip › SUPPLEMENTARY/Figure_S2.pdf]

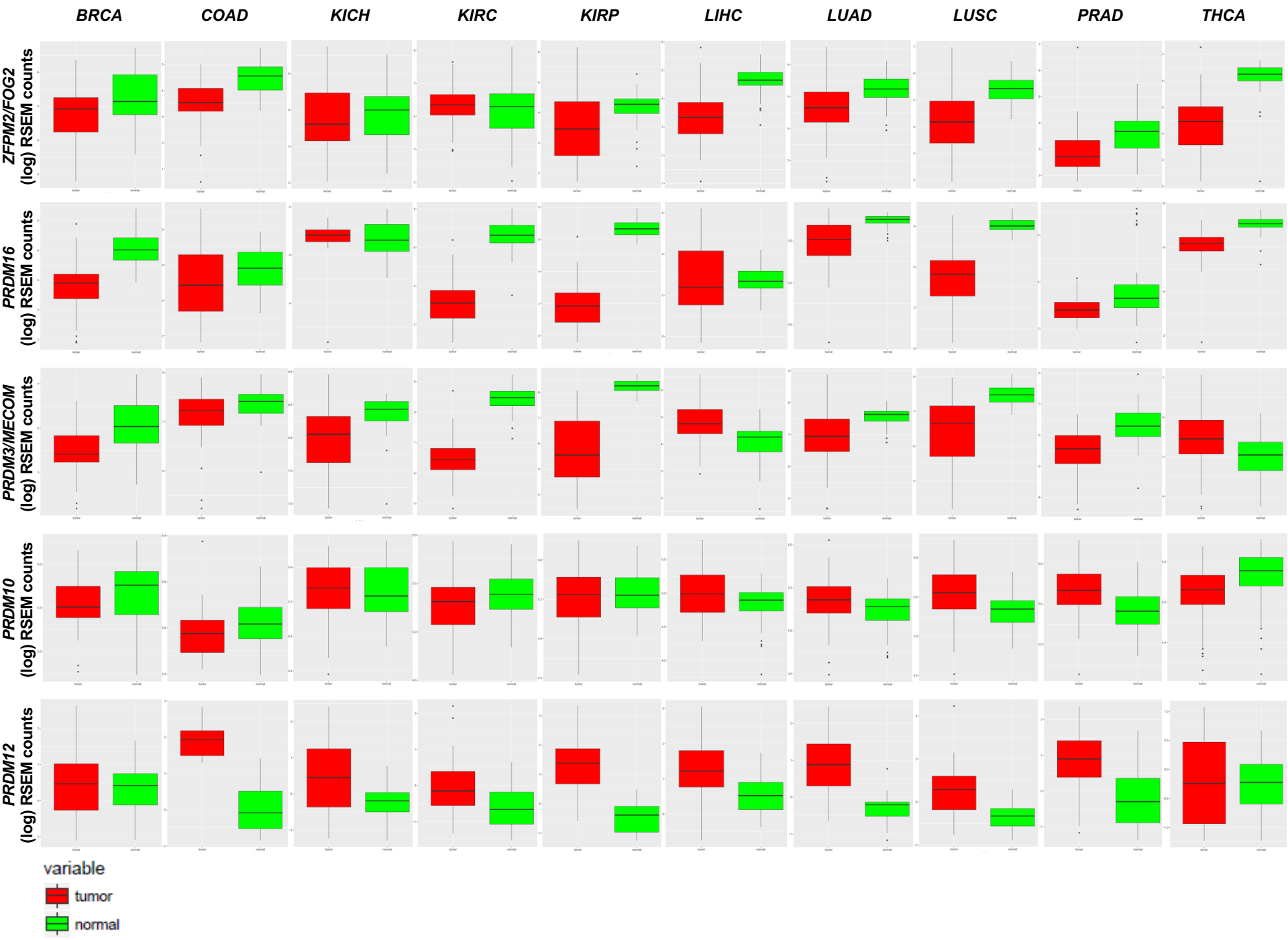

Supplement: Supplementary file 1 [file ijms-19-03250-s001.zip › SUPPLEMENTARY/Figure_S3.pdf]
